# Preparation of a Lotus-Leaf-Inspired PVA–Grape Seed Extract Biomimetic Gel Film and Its Application in Active Packaging for the Postharvest Preservation of *Agaricus bisporus*

**DOI:** 10.3390/gels12100881

**Published:** 2026-09-30

**Authors:** Liyan Wang, Lingyu Sun, Yixuan Zhang, Mengdi Cong, Xin Tan, Lili Ren

**Affiliations:** 1 College of Food Science and Engineering, Jilin Agricultural University, Changchun 130118, China; wangliyan@jlau.edu.cn (L.W.); lingyusun20010416@163.com (L.S.);; 2 Key Laboratory of Bionic Engineering, Ministry of Education, Changchun 130022, China

**Keywords:** lotus leaf-like structure, gel network, antioxidant activity, postharvest preservation

## Abstract

This study reports the fabrication of a biomimetic PVA–GSE film featuring a lotus-leaf-like hierarchical micro/nanostructure, and its application as a gel-based active packaging material for the post-harvest preservation of *Agaricus bisporus* (*A. bisporus*). A lotus-leaf negative template was prepared using soft etching and a polydimethylsiloxane (PDMS) secondary transfer method; the PVA/GSE solution was then cast onto this template and subjected to repeated freeze–thaw cycles for physical cross-linking to induce PVA hydrogelation. The resulting biomimetic gel film reproduces the hierarchical lotus-leaf surface morphology and forms a continuous polymer gel network composed of interconnected gel pores, which serve as reservoirs for grape seed extract (GSE). This gel network structure enhances the properties of PVA. Although BPG-40 exhibited the strongest preservation performance during storage owing to its highest GSE loading, BPG-30 was selected as the optimal formulation, because it achieved the best overall balance of mechanical strength, water-vapour barrier and optical quality while retaining 91–93% of the BPG-40 antioxidant and antibacterial activity; beyond 30% loading, the marginal gains in bioactivity were outweighed by a 12.6% decrease in tensile strength, severe colour darkening (L* = 6.07) and surface cracks observed by SEM. An experiment was conducted to evaluate the effects of the biomimetic composite film on *A. bisporus* over a 12 days storage period at 4 °C. Storage results demonstrate that this film effectively reduces the weight loss rate of *A. bisporus*, lowers their respiration rate, enhances the polyphenol retention rate, and significantly extends the storage life of *A. bisporus*. This study simulates the surface structural regulation mechanism of lotus leaves and represents an innovative approach that synergistically integrates active ingredients with material structure for use in fresh-keeping packaging materials.

## 1. Introduction

Fresh fruits and vegetables are prone to spoilage and quality deterioration owing to inadequate postharvest handling, packaging, storage, and transportation, leading to significant economic losses [1,2,3]. *A. bisporus* is highly nutritious, containing abundant dietary fiber, polysaccharides, proteins, amino acids, and minerals. However, the lack of a protective cuticle makes *A. bisporus* susceptible to physical and microbial damage, resulting in quality degradation [4,5,6]. Therefore, efficient preservation methods are crucial for reducing food waste.

Active food packaging is a critical material for preventing oxidative deterioration of packaged foods. In addition to its traditional functions of sealing and protecting food, active food packaging materials play a pivotal role in extending the shelf life of food by maintaining food quality, preventing food degradation, and avoiding microbial contamination. In recent years, biomimetic micro/nanostructures and natural active compounds have been widely employed in functional food packaging. For instance, Huang et al. developed a lotus leaf-inspired surface with a multi-level hydrophobic morphology to achieve waterproofing and antibacterial properties, thereby extending the shelf life of fruits and vegetables [7]; Zhang et al. demonstrated the potential of biomimetic structures for interface regulation through a hierarchical Polycaprolactone architecture [8]; Wang et al. introduced lemon essential oil into chitosan/corn silk protein films to reduce microbial respiration and inhibit microbial growth; while Kanatt et al. incorporated plant extracts into a PVA/gelatin system to delay spoilage in fish and poultry [9]. Building on these advancements, this study integrates a lotus leaf-inspired structure with PVA as the matrix and grape seed extract (GSE) as the carrier for active substances, advancing the application of functional packaging materials in agricultural product preservation [10]. Among natural surfaces, the lotus leaf was selected as the structural template for three reasons: (i) its hierarchical micro/nano-papillae confer a self-cleaning, low-adhesion surface that can be replicated onto PDMS by soft etching and secondary transfer without harsh chemistry; (ii) the replicated surface topography increases the diffusion path length for water vapour and gases while the film remains thin and flexible enough for practical packaging; and (iii) the micro-textured surface is mechanically compatible with the physically cross-linked PVA gel network, so that surface structuring and bulk loading of GSE can be combined in a single film.

Polyvinyl alcohol (PVA) is widely used in the field of food contact materials due to its ability to form physically cross-linked gels through repeated freeze–thaw cycles, thereby enabling the creation of three-dimensional gel networks with tunable cross-linking density, porosity, and water absorption capacity [11,12]. This type of PVA can serve either as a flexible structural substrate or as a gel reservoir for encapsulating antioxidants (such as flavonoids and polyphenols), thereby optimizing the barrier properties, antioxidant performance, and antibacterial efficacy of the packaging film. Films containing anthocyanins exhibit strong antioxidant activity and effectively inhibit food oxidation [13]. The addition of bioactive compounds such as rutin and ethyl vanillin to PVA/CS films enhances their antibacterial and mechanical properties [14].

Grape seed extract (GSE) is a natural antioxidant and antimicrobial compound derived from grape seeds. GSE primarily contains 15 phenolic compounds, including catechins, flavonols, epicatechins, gallates, and proanthocyanidins, exhibiting antibacterial and antioxidant properties [15,16]. In fact, GSE is a safe additive and has been certified by the U.S. Food and Drug Administration (FDA) [17]. Additionally, it can be used to inhibit microbial growth and potential spoilage of foodborne pathogens in fruits and vegetables.

This study refers to the antibacterial properties of the lotus leaf surface microstructure and loads the active substance GSE onto the biological macromolecule PVA to prepare a new type of antibacterial and antioxidant biomimetic composite film. It is expected to play an important role in the field of food preservation, particularly for the preservation of perishable foods, such as *A. bisporus*, and it offers a novel solution.

## 2. Results and Discussion

### 2.1. Structural Characterization

#### 2.1.1. SEM

**Figure 1:**
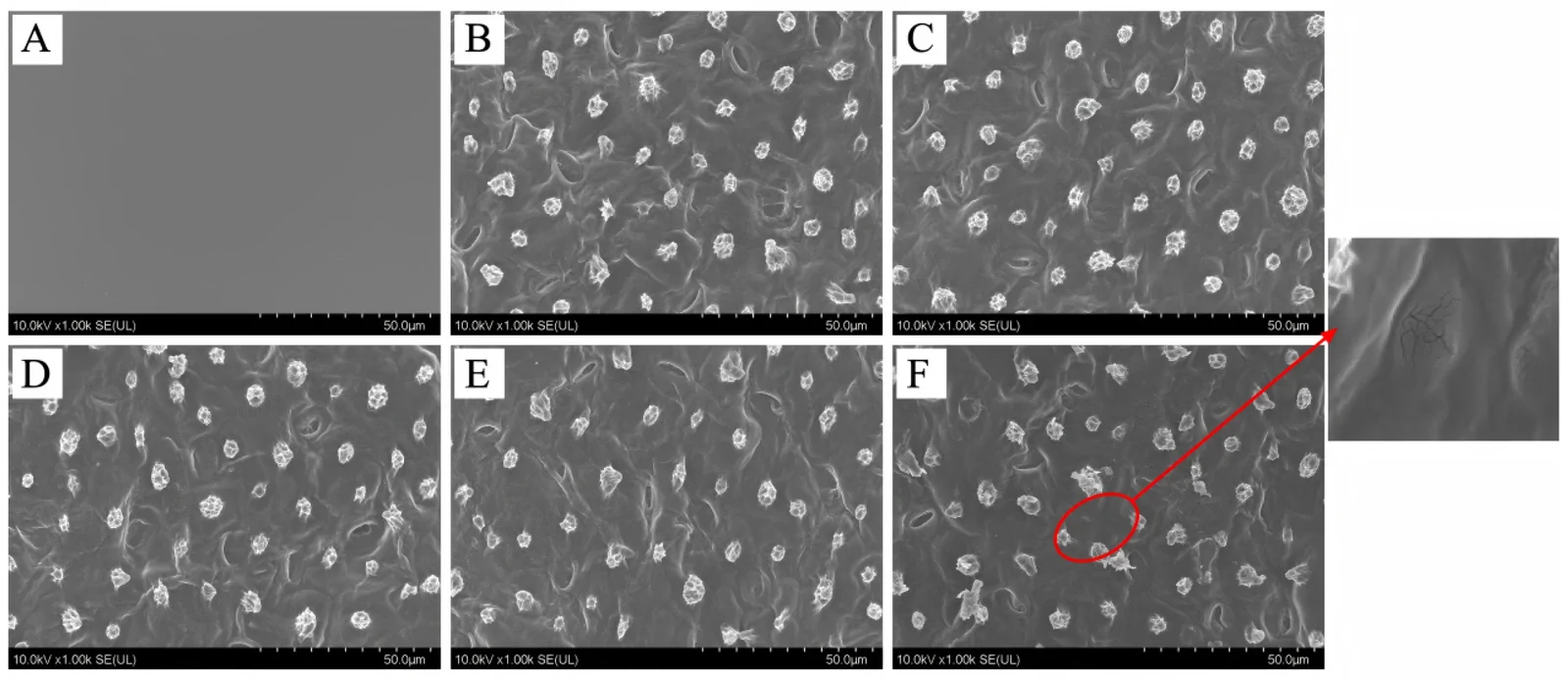
SEM images of GSE–PVA bionic films: (**A**) Control film; (**B**) BPG-0 film; (**C**) BPG-10 film; (**D**) BPG-20 film; (**E**) BPG-30 film; (**F**) BPG-40 film. Data are expressed as mean ± standard deviation (*n* = 3).

The SEM image (Figure 1) reveals the surface morphology of the thin film as well as its gel network structure at the microscopic scale. The control group exhibited a continuous, uniform, smooth, and particle-free surface. In contrast, the BPG film exhibits a rough, lotus-leaf-like surface pattern adorned with numerous white protrusions, indicating that its microstructure has been successfully replicated. The film surface features textured gel domains and interconnected pores, which align with the characteristics of physically cross-linked PVA gels. These gel domains arise from microcrystalline structures and hydrogen-bonded networks formed within the PVA matrix through freeze–thaw cycling; when combined with the lotus-leaf-inspired surface protrusions, they collectively constitute a composite topological-gel structure. This structure holds promise for increasing surface area, enhancing hydrophobicity and barrier properties, thereby helping to reduce water migration and gas exchange and improving post-harvest preservation performance. No significant differences in surface structure were observed among BPG-0, BPG-10, BPG-20, and BPG-30 owing to the enhanced solubility of GSE in PVA at low concentrations. As the GSE content increased, GSE particles tended to agglomerate, causing cracks on the BPG-40 surface and a corresponding decrease in TS. However, the EAB value continued to increase, suggesting that EAB has an effect on the mechanical properties relevant to food preservation. These findings are consistent with the mechanical property analysis.

**Figure 2:**
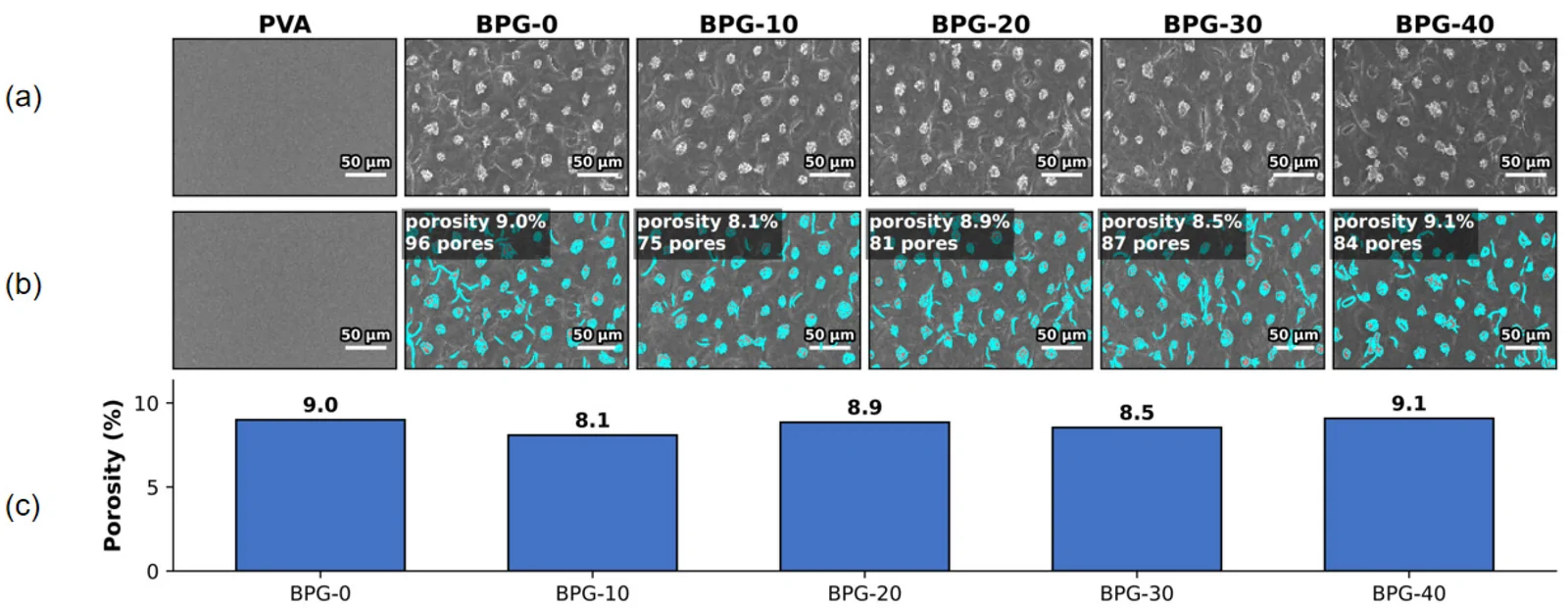
SEM morphology and image-based pore analysis of the PVA control and the BPG films. (**a**) original SEM images; (**b**) segmentation overlay (cyan contours) from Otsu thresholding; scale bar = 50 μm. The PVA control lacked sufficient contrast for reliable segmentation and is shown for qualitative morphology only. (**c**) 2D surface porosity (%) of the BPG films; pore counts per field are labelled on the overlays. Data are from one representative SEM field per formulation. Notes: Scale bar = 50 µm, consistent with the SEM instrument watermark (×500, 50.0 µm). Pores were segmented by Otsu thresholding on the grayscale SEM images; the minority phase was taken as the pore class, and objects < 80 px were excluded as noise. The PVA film showed low SEM contrast and was therefore not reliably quantifiable (morphology only).

Quantitative image analysis of the SEM micrographs (Otsu segmentation on grayscale images; scale bar = 50 μm; Figure 2) was performed on the biomimetic BPG films. The pure PVA control film showed a low-contrast, relatively featureless surface under SEM that prevented reliable automated pore segmentation, so it is presented for qualitative morphology only (Figure 2a). The BPG films exhibited low surface porosity (8.1–9.1%; 75–96 pores per field), with mean equivalent pore diameters of 7.4–8.1 μm, indicating that casting on the lotus-leaf-templated PDMS surface produces a consolidated surface layer atop the gel network. Among the BPG films, BPG-30 showed the most homogeneous pore-size distribution (SD 3.78 μm), consistent with its optimal mechanical integrity, whereas BPG-40 showed a slight porosity rebound (9.1%) associated with GSE agglomeration and the surface cracks visible in Figure 1F. These interconnected ~2–16 μm gel pores plausibly act as reservoirs for GSE and provide water-filled channels for its sustained release (see Section 2.6.3).

#### 2.1.2. FTIR

**Figure 3:**
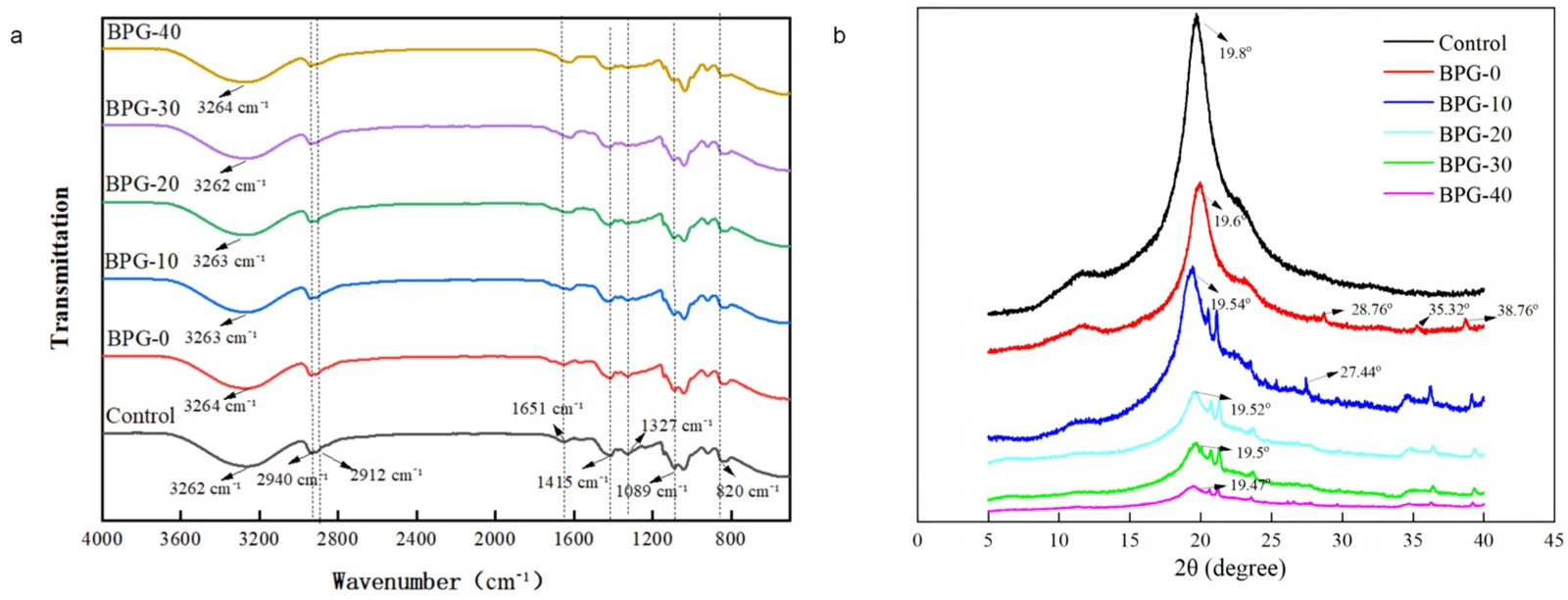
(**a**) FTIR spectra of the control, BPG-0, BPG-10, BPG-20, BPG-30, and BPG-40 film. (**b**) XRD patterns of the control, BPG-0, BPG-10, BPG-20, BPG-30, and BPG-40 film. Data are expressed as mean ± standard deviation (*n* = 3).

The FTIR infrared spectra of control, BPG-0, BPG-10, BPG-20, BPG-30, and BPG-40 films (Figure 3a) revealed an absorption band at 3262 cm^−1^, corresponding to –OH stretching vibrations. Peaks at 1327 cm^−1^, 2940 cm^−1^, and 2912 cm^−1^ were attributed to C–H stretching vibrations. Peaks at 1089 cm^−1^ corresponded to C–O–C stretching vibrations [18,19,20,21]. With increasing GSE content, the intensity of the 1415 cm^−1^ peak in the BPG films gradually increased compared with the control and BPG-0 films. However, the BPG films underwent only minimal deformation. This indicates that the addition of GSE enhanced intermolecular bonding on the film surface. Additionally, the C–C vibration peak in the GSE–PVA films shifted from 1653 cm^−1^ to 1609 cm^−1^ compared with the control film peak at 1654 cm^−1^. This shift was attributed to increased stretching of hydroxyl groups, suggesting the formation of hydrogen bonds between GSE and PVA. Overall, the FTIR results confirmed that GSE significantly improved the compatibility of PVA and strengthened intermolecular interactions, consistent with the findings reported by Li and Zhang et al. [22].

#### 2.1.3. XRD

Figure 3b shows the XRD patterns of the control and biomimetic films. The XRD pattern of the highly crystalline PVA displayed sharp diffraction peaks at 2θ = 19.8° [23]. In contrast, the XRD pattern of BPG-0 (Figure 3b) revealed new crystalline peaks at 2θ = 28.76°, 35.32°, and 38.76°. This indicates that the lotus leaf-like mimetic surface enhanced film crystallinity [24]. With the incorporation of GSE into PVA, the characteristic peak at 2θ = 19.8° shifted to lower angles. Moreover, the peak intensities at θ = 28.76°, 35.32°, and 38.76° first increased and then decreased, indicating the formation of hydrogen bonds between the hydroxyl groups of PVA and the polyphenols in GSE. However, the peak intensities at 2θ = 27.44° for BPG-20, BPG-30, and BPG-40 decreased owing to the uneven dispersion of GSE in PVA caused by the biomimetic structures.

### 2.2. Physical Properties of Composite Films

#### 2.2.1. Color Analysis of Films

**Figure 4:**
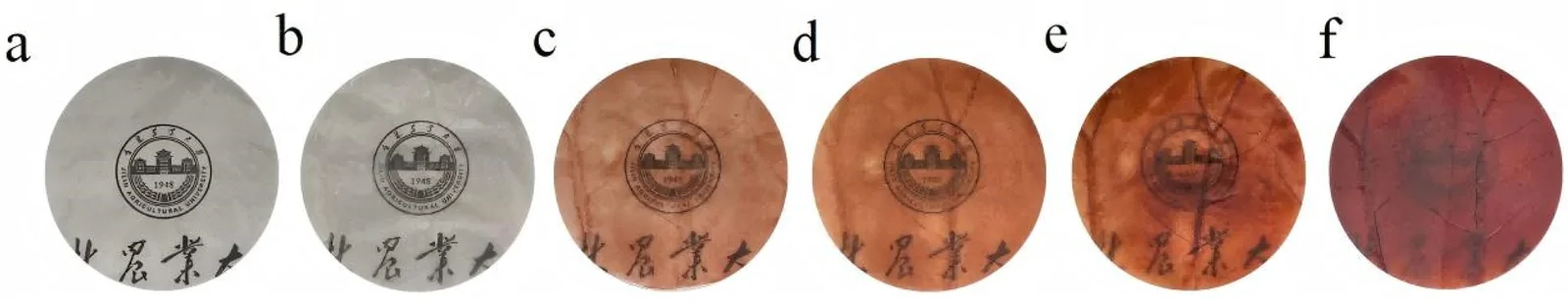
Visual appearance of PVA-GSE bionic films: (**a**) Control; (**b**) BPG-0; (**c**) BPG-10; (**d**) BPG-20; (**e**) BPG-30; (**f**) BPG-40. Note: The dark coloration of BPG-40 (L = 6.07) results from the high loading of grape seed extract (40%), which imparts its natural brown-purple pigment to the PVA matrix. Data are expressed as mean ± standard deviation (*n* = 3).

Color is a key indicator of film packaging quality and affects the visual appeal of food. The visual maps of each film group are shown in Figure 4, and the corresponding color measurements are presented in Table 1. Both the control and BPG-0 films were colorless and transparent. This indicates that the biomimetic structure did not alter the film color [25,26,27]. With increasing GSE content, the L* and b* values of the biomimetic film decreased, while the a* and ΔE values considerably increased [28]. The decrease in the L* value was attributed to interactions between PVA and GSE, which darkened the film and reduced its transparency, thereby effectively blocking ultraviolet light. The decrease in the b* value indicated a gradual fading of the yellow hue, while the increase in the a* value suggested a shift toward a reddish-brown tone. ΔE represents the overall color difference in the film and is negatively correlated with L*. As a* increased and b* decreased, ΔE significantly increased, reflecting a pronounced color change, consistent with the visual appearance of the packaging film (Figure 4).

The progressive reddish-brown darkening with increasing GSE content arises from the intrinsic chromophores of GSE. Oligomeric proanthocyanidins and anthocyanidin units (e.g., cyanidin and delphinidin) in grape seed extract absorb strongly in the 500–540 nm region, imparting red-brown hues whose intensity increases with pigment loading. During film drying, partial oxidation of catechin and epicatechin units to the corresponding o-quinones further deepens the brown tone. In addition, at high GSE loadings the phenolic chromophores self-associate within the PVA matrix (copigmentation and intermolecular π–π stacking), which intensifies and bathochromically shifts the visible absorption; this is reflected in the monotonic decrease in L* (from 83.29 for BPG-0 to 6.07 for BPG-40) and the increase of a* (from 1.52 to 15.48). From an application perspective, this darkening attenuates light transmission (beneficial for retarding light-induced oxidation of the packaged mushroom) but compromises the visual transparency expected of display packaging.

#### 2.2.2. Analysis of Thickness and Barrier Properties

Film thickness is a key parameter influencing barrier properties. The control and BPG-0 films had thicknesses of 0.13 mm and 0.24 mm, respectively (Table 2). The addition of GSE increased the thickness of the biomimetic films, ranging from 0.36 mm to 0.46 mm. This indicates that the increase in film thickness is jointly driven by the plasticizing expansion of the PVA matrix and the structural occupation induced by the loading of GSE within the hydrogel pores; this effect promotes the outward swelling of the network from a mechanical perspective [29]. Previous studies have shown that incorporating carboxymethyl CS into black carrot powder to replicate a lotus leaf-like biomimetic surface resulted in thicker films [24].

We measured moisture content (MC), water vapour permeability (WVP), swelling rate, and solubility. Among the biomimetic films (control, BPG-0, BPG-10, BPG-20, BPG-30, and BPG-40), the control group exhibited the highest MC at 18% (Table 2). This was due to hydrogen bonding between water molecules and hydrophilic hydroxyl groups. The biomimetic structure reduced the MC of the BPG-0 film owing to its increased surface area. The incorporation of GSE further reduced the MC of the BPG biomimetic films. This may be attributed to GSE limiting hydrogen bonding with water molecules. Similarly, a reduction in the MC of carboxymethyl cellulose films containing gamboge anthocyanin [30].

Swelling rate and solubility are key factors influencing the suitability of food packaging materials and serve as indicators of film stability [31]. The control film had a low swelling rate of 517.88%. The incorporation of the biomimetic structure and GSE increased the swelling rates of the films (Table 2). This phenomenon is attributed to an increase in the film’s structural porosity and a reduction in the effective crosslinking density, thereby promoting more pronounced swelling [32]. Similarly, the incorporation of a biomimetic structure and the elevation of GSE concentration also enhanced the water solubility of the film, reflecting the partial dissolution or plasticization effect of low-molecular-weight phenolic compounds on the gel phase and the disruption of the film’s intact network structure. This phenomenon is consistent with the variation in thin-film thickness.

**Figure 5:**
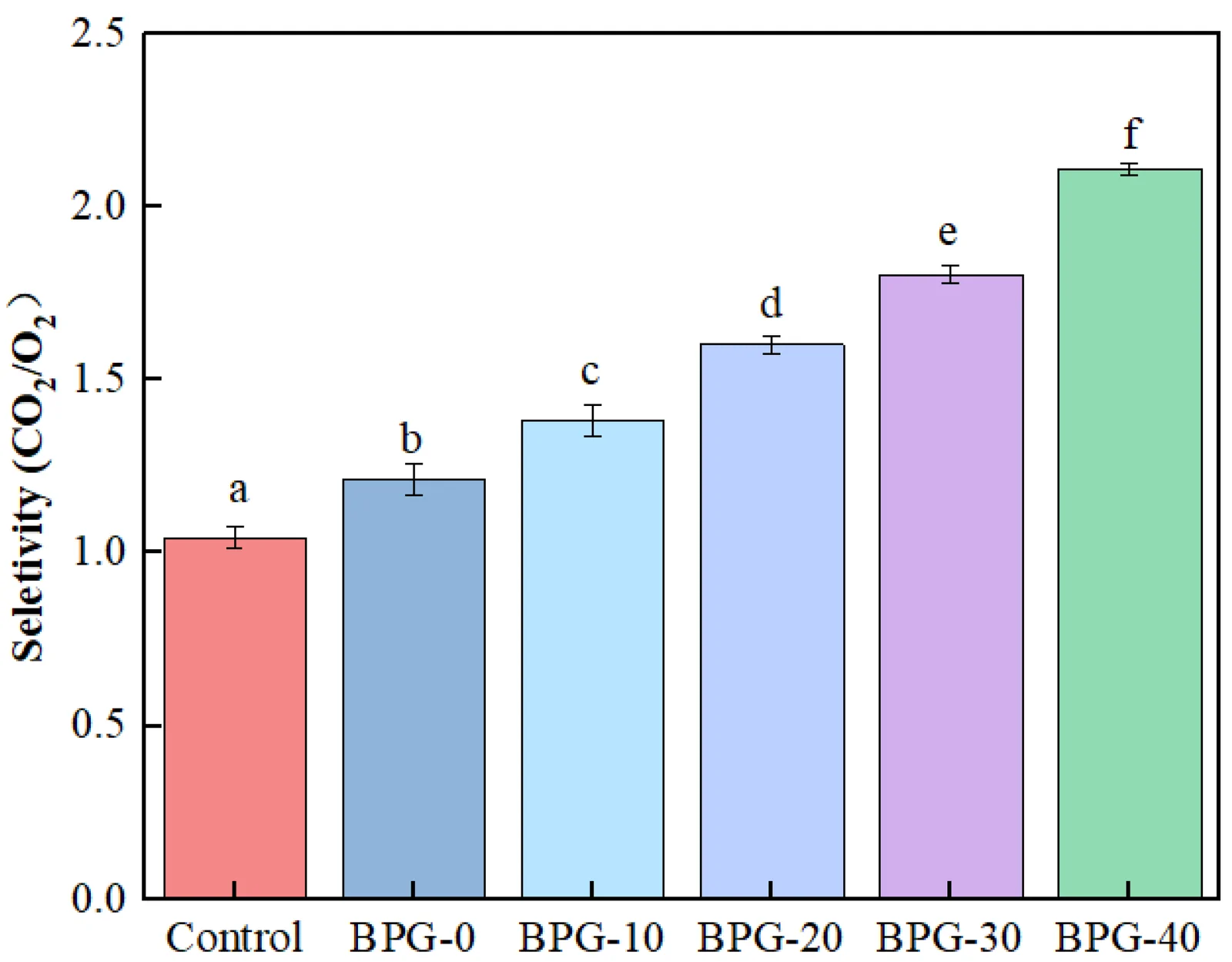
CO_2_/O_2_ selectivity of the control, BPG-0, BPG-10, BPG-20, BPG-30, and BPG-40 film. Data are expressed as mean ± standard deviation (*n* = 3). Different lowercase letters indicated significant differences among groups (*p* < 0.05).

Gas permeability is a key property for food packaging applications [33]. The synergistic interaction between the lotus leaf morphology and the underlying gel network creates a tortuous diffusion pathway, thereby altering the transport behavior of water vapor and gases. The control film displayed the lowest CO_2_/O_2_ selectivity value (1.04 ± 0.03) and the highest WVP (9.47 × 10^−10^ ± 1.39 g m^−1^ s^−1^ Pa^−1^), respectively (Figure 5, Figure S4 and Table 2). In contrast, the biomimetic film demonstrates a significantly reduced WVP value and enhanced gas selectivity. This improvement is likely attributable to the crosslinking density and hydrophilic properties of the gel network, which determine both the water adsorption capacity and the effective free volume available for diffusion: a denser gel network, with its more interconnected pores, reduces the WVP value by limiting water absorption and forming narrower diffusion channels; whereas a more open gel pore structure enhances transient water transport capability. Additionally, this hierarchical surface structure increases the diffusion path length and impedes direct water vapor flux, while the interaction between the gel and polar gases (e.g., carbon dioxide) can modulate the CO_2_/O_2_ selectivity. The apparent CO_2_/O_2_ selectivity is more plausibly explained by a solution–diffusion mechanism: CO_2_ is considerably more soluble than O_2_ in the polar, hydrogen-bonding PVA–GSE matrix, which enhances its permeability through the hydrated gel network, whereas the reduced free volume of the denser network restricts O_2_ transport. This permeation behaviour tends to reduce the availability of O_2_ inside the package, thereby increasing the CO_2_/O_2_ selectivity ratio. The film’s air permeability increases gradually as the GSE content rises. Due to the polar nature of CO_2_, its carbon atoms can interact with the oxygen atoms in the GSE, thereby facilitating the rapid penetration of the gas through the film; in contrast, O_2_ is a non-polar gas, and its permeability depends primarily on the porosity of the film. As a result, the WVP value decreases. When the GSE content reaches the level observed in the BPG-40 group, the excess GSE forms localized aggregates through intermolecular interactions, thereby disrupting the continuity of the substrate and creating new permeation pathways. This leads to a slight increase in the WVP value to 4.87 × 10^−10^ g·m^−1^·s^−1^·Pa^−1^ [34]. Similarly, Qin et al. [35] reported that the addition of betaine from pitaya skin to PVA/starch films reduced WVP. This effect is similar to the hydrogen bonding between GSE and the PVA matrix, which influences the film structure.

#### 2.2.3. Mechanical Properties

Tensile strength (TS) and elongation at break (EAB) are vital indicators for evaluating the mechanical properties and preservation of films. The control and BPG-0 biomimetic films exhibited TS values of 23.09 MPa and 19.13 MPa, respectively (Table 3). The BPG-0 film exhibited a significantly lower TS than the control group because the biomimetic structure reduced film strength. With increasing GSE content, the TS of the biomimetic films first increased and then decreased.

When the GSE content is 30%, hydrogen bonds form between the hydroxyl groups of PVA and the polyphenolic groups in GSE, promoting more compact molecular ordering and generating strong interfacial adhesion; additionally, the macromolecular components of GSE entangle with the PVA chains, resulting in physical cross-linking. These crosslinked structures reinforce the gel network and enhance its TS. The synergistic effect of these two mechanisms enhances intermolecular cohesion, leading to a sustained increase in TS. However, when the GSE content reaches 40%, the aggregation of excess GSE may reduce the homogeneity of the matrix and lower the TS; however, GSE can also disrupt the intermolecular hydrogen-bond network of PVA and increase chain mobility, thereby further improving EAB. This reduced the effective interaction between GSE and PVA and formed inhomogeneous phase regions that served as stress concentration points, disrupted the original matrix structure, and reduced TS to 35.67 ± 0.40 MPa. This trend is consistent with previous research findings, highlighting the critical role of the gel network structure between the additive and the matrix in determining the macroscopic properties of composite materials. This trend is consistent with previous studies and highlights the key role of intermolecular forces between additives and matrices in determining the macroscopic properties of composites [36]. Similarly, the addition of polygonum multiflorum polysaccharide modified the TS of polyvinyl alcohol-based films [37].

Additionally, the control and BPG-0 films had EAB values of 33.31% and 36.93%, respectively (Table 3). The EAB of the biomimetic film gradually increased with increasing GSE content. The phenolic hydroxyl, carboxyl, and other functional groups in GSE competed with the hydroxyl groups on PVA chains to form hydrogen bonds. This competition disrupted the original strong hydrogen bond network of PVA, reduced intermolecular bonding, and facilitated the movement of chain segments. Moreover, the hydrogen bonds between GSE and PVA altered the internal spatial structure of the films, further enhancing their flexibility [38].

TS represents the ultimate tensile strength of the films at tensile failure and reflects the basic stress requirements for food packaging and transport. EAB indicates film flexibility and influences overall mechanical properties. The EAB of artificial fibers is particularly sensitive to changes in mechanical performance [39]. Therefore, the observed optimal performance at 30% GSE (BPG30) reflects a balance between the cohesive strength of the gel network and the mobility of the chain segments, thereby yielding excellent gel strength and flexibility suitable for packaging applications.

### 2.3. Antioxidant Activity

Antioxidant activity is crucial for prolonging food shelf life and inhibiting spoilage. Therefore, developing food packaging films with antioxidant properties is vital for slowing oxidation and maintaining food quality and freshness. The 2,2-diphenyl-1-picrylhydrazyl (DPPH) and 2,2′-azino-bis(3-ethylbenzothiazoline-6-sulfonic acid) (ABTS) radical scavenging rates and ferric reducing antioxidant power (FRAP) values of BPG-0 film slightly increased to 3.70%, 9.31%, and 0.50 mg TE/g, respectively, compared with the control film (Table 4). This enhancement was attributed to the biomimetic structure. Overall, this biomimetic film structure exhibits higher antioxidant activity than traditional composite films, this is because its biomimetic design incorporates a mesh-like gel structure that physically embeds the GSE within the gel pores and enhances active substance retention through hydrogen bonding interactions, thereby improving its antioxidant performance [24]. The scavenging ability of the films for DPPH and ABTS radicals increased with increasing GSE content. The DPPH and ABTS radical scavenging rates and ferric ion reducing ability of the BPG-40 films reached 64.18%, 65.24%, and 1.83 mg TE/g, which were 18, 8, and 4 times those of the control films. These results are consistent with previous studies, confirming that the polyphenols in GSE effectively scavenge free radicals through hydrogen atom and electron donation.

### 2.4. Antibacterial Activity

**Figure 6:**
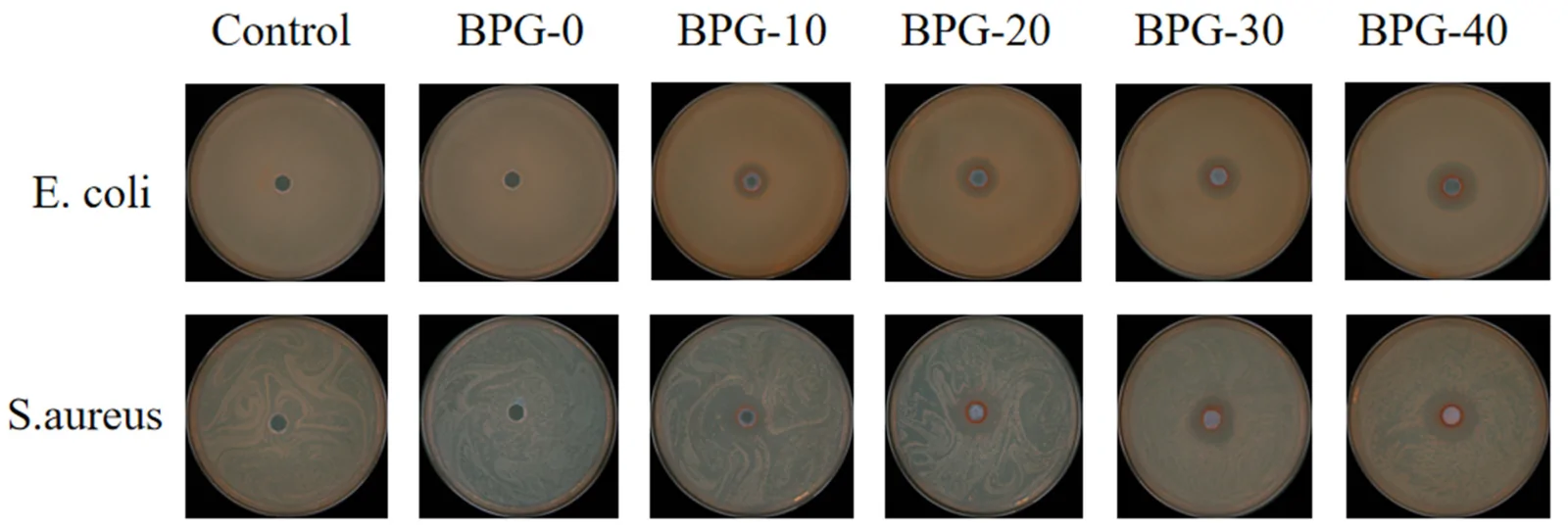
The antibacterial activity of the Control, BPG-0, BPG-10, BPG-20, BPG-30, and BPG-40 against various pathogens.

Microbial invasion is a key factor contributing to food spoilage. As shown in Figure 6, the antimicrobial properties of GSE–PVA biomimetic films were evaluated using the zone of inhibition method against *E. coli* and *S. aureus*. The control and BPG-0 films exhibited negligible inhibition zones against *E. coli* and *S. aureus*, indicating weak antimicrobial activity (Table 5). Previous studies have shown that pure PVA films exhibit minimal antibacterial activity, and their inhibitory effects mainly depend on physical barrier properties [40]. Lotus leaf-like biomimetic films exhibited weak antibacterial performance owing to their ability to prevent bacterial attachment rather than directly killing bacteria [41]. However, the GSE–PVA biomimetic films displayed significant antibacterial activity. As the GSE content increased from 10% to 40%, the inhibition zones of the biomimetic films increased. This indicates that GSE effectively enhances the antimicrobial activity of biomimetic films. Therefore, pure PVA films or biomimetic structures alone have limited antibacterial effects. The combination of GSE and the biomimetic structure significantly improves the antibacterial performance of the films.

### 2.5. Application

#### 2.5.1. Physical Characteristics of *A. bisporus*

**Figure 7:**
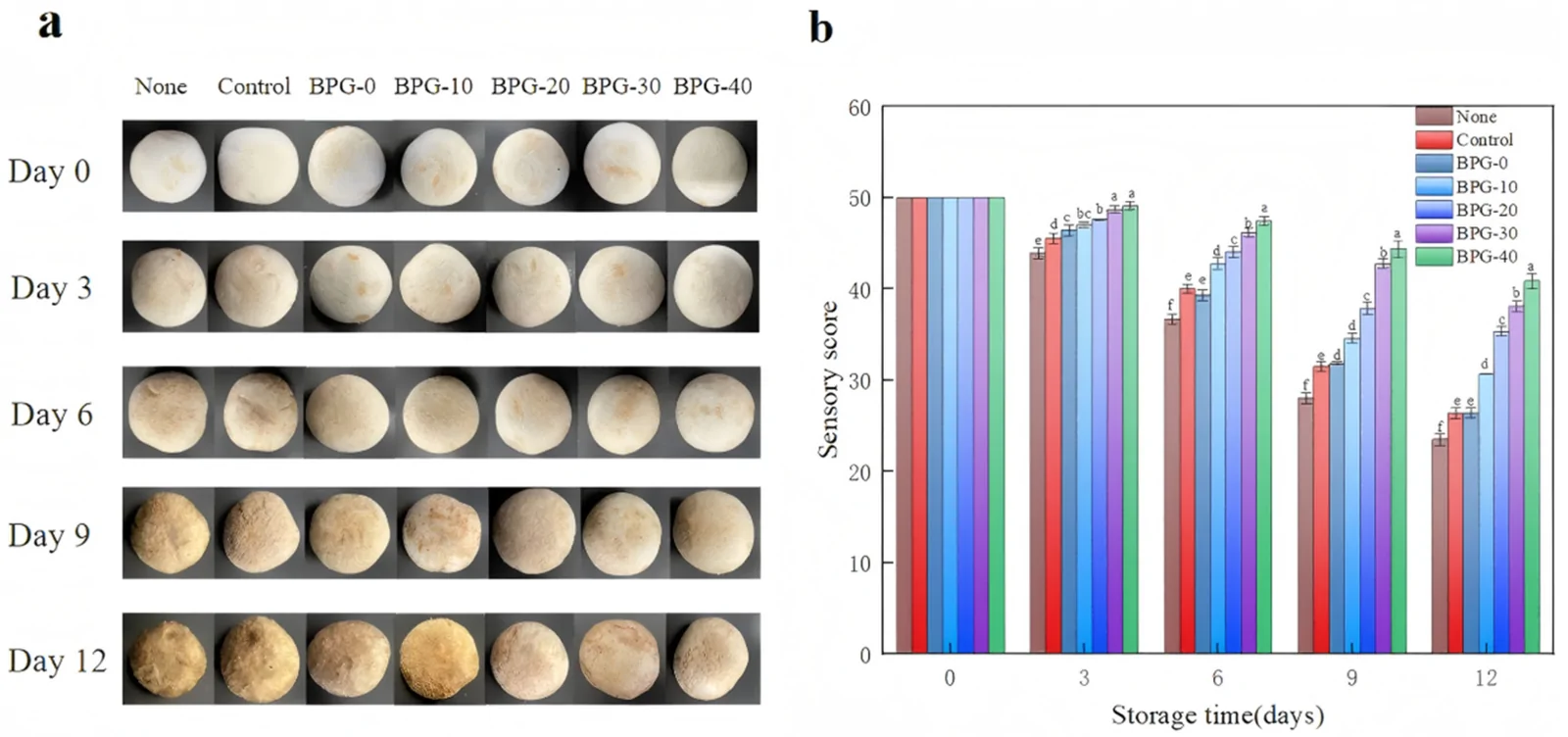
(**a**) Changes in the appearance of *Agaricus bisporus* under direct air exposure and in different packaging films during storage (day 0–12). (**b**) Sensory evaluation of *Agaricus bisporus* over the 12-day storage period. Data are expressed as mean ± standard deviation (*n* = 3). Different lowercase letters indicated significant differences among groups (*p* < 0.05).

Improper storage can lead to vegetable deterioration, which reduces their market value. To evaluate the preservation performance of GSE–PVA films on *A. bisporus*, seven groups of fresh *A. bisporus* were wrapped in the films (Figure 7). These films provide antioxidant and antimicrobial properties and may reduce the respiration rate, potentially extending the shelf life of *A. bisporus*. The storage conditions of *A. bisporus* under different film wrappings over 12 days are shown in Figure 7a. By day 9, unwrapped *A. bisporus* exhibited rotting, browning, and wilting. As the GSE content increased, wrapped *A. bisporus* maintained a natural appearance with reduced rotting. The BPG-40 film exhibited the highest preservation performance. By day 12, most samples exhibited significant spoilage and browning. However, the high-dose BPG group retained the overall morphology of *A. bisporus* compared with the other groups. Additionally, consumers evaluated the effects of each film on colour, taste, texture and overall acceptability (Figure 7b). On day 0, all samples exhibited good overall appearance, uniform color, normal aroma, and firm texture. The corresponding sensory scores were around 50, which was significantly above the 30-point threshold, indicating high consumer acceptance. By day 12, the unwrapped samples exhibited significant deterioration, characterized by dull colors, loss of aromas, and soft textures, with sensory scores below 30 points. In contrast, *A. bisporus* wrapped in the BPG-40 film maintained its color, aroma, and texture, achieving sensory scores above 40 points. Research has indicated that appropriate preservation treatments can preserve the color, aroma, and texture of food through the inhibition of oxidation and microbial growth, thereby enhancing sensory quality and consumer acceptance [42]. The performance of the BPG film series in this experiment was consistent with this preservation mechanism, highlighting their potential to extend shelf life and maintain sensory quality. Among all samples, the BPG-40 film achieved the highest sensory scores. This indicates that the high GSE–PVA dosage effectively preserved the sensory properties of *A. bisporus* and improved consumer acceptance.

Note that BPG-30 was identified as the optimal film formulation based on a multi-response balance of ten film-property indicators (see Section 2.6.2 for details), whereas the storage experiment was designed to compare all formulations under identical conditions; the superior storage performance of BPG-40 therefore reflects its higher GSE loading rather than superior film quality.

#### 2.5.2. *A. bisporus* Preservation

**Figure 8:**
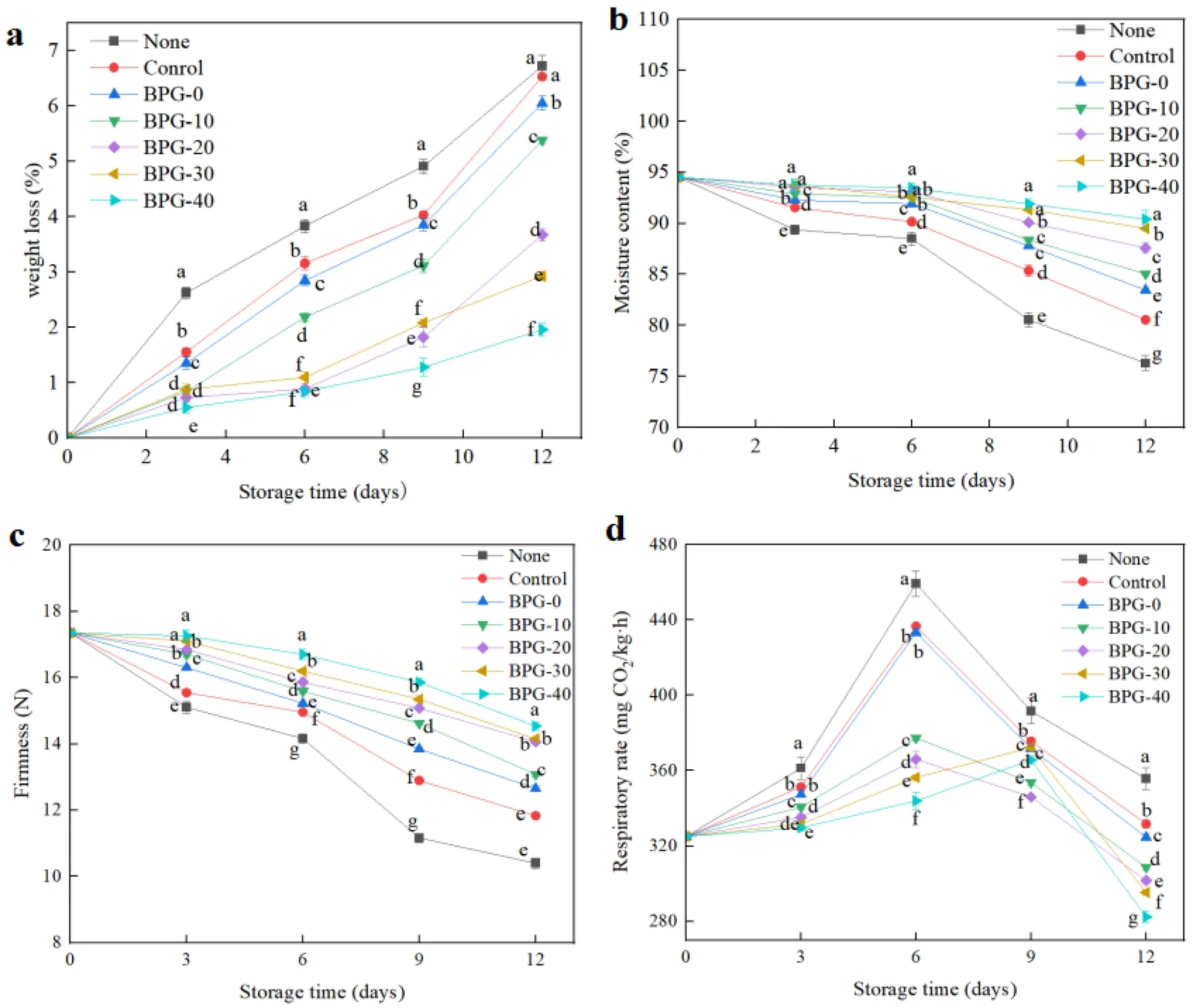
Changes in (**a**) weight loss, (**b**) MC, (**c**) respiration rate, (**d**) firmness of *Agaricus bisporus* during 12 d of storage in the unwrapped, BPG-0, BPG-10, BPG-20, BPG-30, and BPG-40 groups. Data are expressed as mean ± standard deviation (*n* = 3). Different superscript letters at the same storage time indicate significant differences (*p* < 0.05), as analyzed by one-way ANOVA with Tukey’s HSD or Tamhane’s T2 post hoc test. Data are expressed as mean ± standard deviation (*n* = 3).

During extended storage, MC decreased, and weight loss increased owing to cell damage and water migration, which accelerated quality deterioration, including wrinkling and shrinkage. The weight loss rate of all *A. bisporus* groups gradually increased over time (Figure 8a). Notably, all film-wrapped groups consistently exhibited lower weight loss rates than the unwrapped group throughout the storage period. On day 12, the unwrapped group exhibited a high weight loss rate (6.72%) than the control (6.58%) and BPG-0 (6.05%), BPG-10 (5.38%), BPG-20 (3.67%), BPG-30 (2.92%), and BPG-40 (1.95%) groups.

The hydrophobicity of the biomimetic structure significantly improved the moisture resistance of the packaging, thereby reducing the weight loss of *A. bisporus.* The BPG-40 group provided the most effective packaging, indicating that a higher GSE content in the GSE–PVA film improved moisture retention and preserved the freshness of *A. bisporus*. After 12 days of storage, the MC of *A. bisporus* in the control and BPG-0 groups was 6% and 9% higher than that of the unwrapped group, respectively. The BPG-40 group consistently maintained the highest MC (Figure 8b), consistent with the weight loss trends shown in Figure 8a. Similarly, potato starch films incorporated with cinnamon essential oil reduced water loss in *A. bisporus* [43].

The rate of weight loss in fruits and vegetables is closely related to water loss and respiration rate [44]. Weight losses exceeding 4–6% caused wilting or significant surface wrinkling. The respiration rate of *A. bisporus* first increased and then decreased over 12 days of storage (Figure 8c). The incorporation of biomimetic structures and the interaction between GSE and the PVA matrix modified the film structure. This modification significantly reduced WVP, minimized moisture fluctuations within the package, and alleviated weight loss caused by moisture evaporation. Furthermore, the gas permeability of the film enhanced CO_2_/O_2_ selectivity, creating a low-O_2_ and high-CO_2_ microenvironment that may inhibit respiratory rates. Notably, the BPG-30 and BPG-40 films were most effective at delaying the respiration peak, extending it from 6 to 9 days. Similarly, *A. bisporus* packaged with bioactive CS/zein film containing α-tocopherol exhibited a delayed respiration peak [45]. By day 12, the respiration rate of some BPG groups decreased below their initial levels. The respiratory rate of the BPG40 group on day 12 was lower than the initial value, which can be attributed to the film’s excellent permeability to O_2_ and CO_2_. Moreover, the gas-selective permeability of the film and WVP regulation contributed to delaying the respiration peak and stabilizing the internal packaging microenvironment.

The hardness of all *A. bisporus* groups decreased during storage (Figure 8d). On day 12, the hardness of the control and BPG-0 groups was 11.34% and 21.63% higher than that of the unwrapped group, respectively. The BPG-40 group reached a hardness of 15 N, which was 40% higher than the unwrapped group. This indicates that the antioxidant activity of GSE and the antimicrobial properties of the biomimetic films effectively limited oxygen and microbial invasion, reduced WVP, and prevented *A. bisporus* degradation. Consequently, the respiration rate and water loss of *A. bisporus* decreased, thereby delaying the reduction in *A. bisporus* hardness. Hydroxypropyl methyl cellulose/kudzu-modified cling films more effectively preserved the hardness of *A. bisporus* than the control film [5].

#### 2.5.3. Analysis of Antioxidant Properties

**Figure 9:**
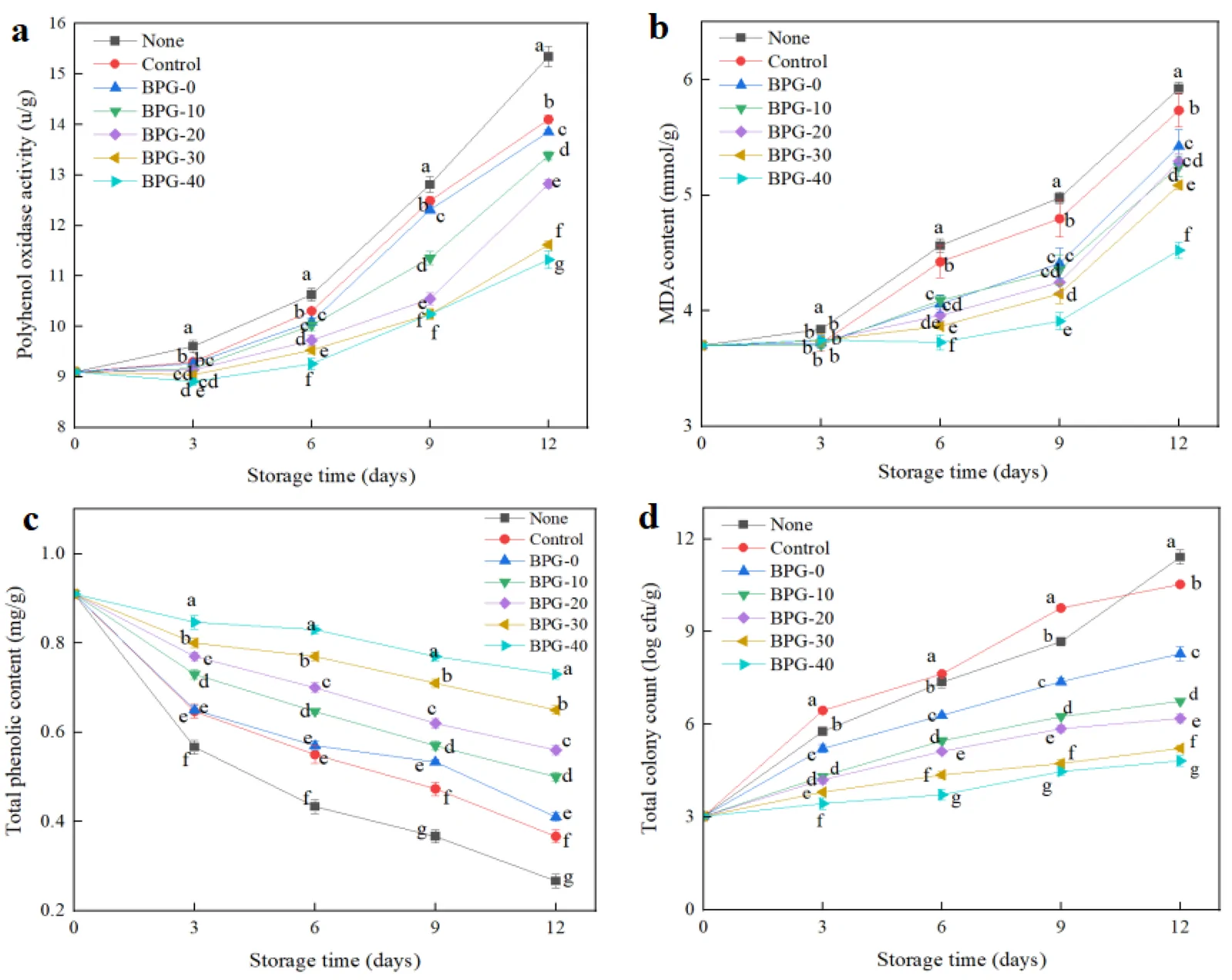
Effects of different packaging materials on (**a**) PPO activity, (**b**) MDA content, (**c**) TPC, and (**d**) total bacterial count of *Agaricus bisporus* during a 12-day storage period. Data are presented as means ± SEM (*n* = 3). Different superscript letters at the same storage time indicate significant differences (*p* < 0.05), as determined by one-way ANOVA with Tukey’s HSD or Tamhane’s T2 post hoc test. Data are expressed as mean ± standard deviation (*n* = 3).

Polyphenol oxidase (PPO) drives enzymatic browning in fruits and vegetables, and its activity is a key indicator for assessing the aging and quality changes in *A. bisporus* [46]. During the 12-day storage period, PPO activity in all treatment groups significantly increased over time (Figure 9a). However, samples packaged with the BPG-40 film consistently exhibited the lowest PPO activity. As the GSE content increased, the film permeability to O_2_ increased, thereby limiting the contact between *A. bisporus* and O_2_ and inhibiting enzymatic browning. Moreover, the stable mechanical properties of the film supported its OP regulation, thereby ensuring consistent gas barrier performance throughout the storage and preventing preservation failure from mechanical damage. The slower color change of *A. bisporus* (Figure 7a) further confirmed the antioxidant performance and mechanical stability of the composite film, as reflected in its appearance quality.

Malondialdehyde (MDA) is a key biochemical indicator of lipid peroxidation in fruits and vegetables, and its content is positively correlated with cell membrane damage and tissue aging [47]. The increase in MDA content was significantly slower in the BPG-40 packaging group compared with the other groups (Figure 9b). In contrast, the MDA content in the unwrapped group continued to increase. Films containing GSE and biomimetic structures effectively reduced MDA accumulation, thereby inhibiting lipid peroxidation and tissue aging. This trend is consistent with the PPO activity changes shown in (Figure 9a), further confirming the antioxidant role of GSE-containing films. This effect may be attributed to the excellent mechanical properties of the film, which prevent abnormal MDA accumulation from film damage, delay tissue aging in *A. bisporus*, and inhibit quality deterioration. Similarly, anthocyanins in CP–FA films can reduce MDA content and inhibit lipid oxidation, thereby effectively extending the shelf life of *A. bisporus* [22].

Phenolic compounds are vital physiological bioactive components in *A. bisporus*. Total phenolic content (TPC) typically increases during maturation but decreases with aging and browning [48]. (Figure 9c) shows the changes in TPC of *A. bisporus* under different film packaging treatments. The results revealed that the BPG-40 packaging group consistently maintained higher TPC than the other groups throughout storage.

This effect can be attributed to the superior TS of the BPG-40 film, which effectively prevents film expansion or rupture caused by gas production during the respiration of *A. bisporus* and minimizes cellular damage from packaging. Pineapple core extract can increase the TPC of apples, likely because fruits accumulate phenolic compounds as a self-protection response to exogenous stimuli and promote cell repair [46].

#### 2.5.4. Total Colony Counts Analysis

The high MC and neutral pH of *A. bisporus* provide favorable conditions for microbial growth. Bacterial infection accelerates *A. bisporus* aging and reduces its quality, while film packaging can provide protective effects. (Figure 9d) shows the total colony counts of *A. bisporus* packaged with different films. Throughout the storage period, colony counts increased in all treatment groups. The unwrapped group had the highest counts, while the BPG-40 packaging group exhibited the lowest counts. This effect can be attributed to the strong antibacterial properties of GSE. Furthermore, the biomimetic structure may increase the contact area between the film and the surface of the double-spore *A. bisporus*, thereby promoting extract release and enhancing the synergistic effect of physical inhibition of bacterial attachment and antibacterial components.

The variations in PPO activity (Figure 9a), MDA content (Figure 9b), and TPC (Figure 9c) indicate the excellent antioxidant properties of the film. These properties can reduce cellular oxidative damage, maintain the structural integrity and physiological functions of *A. bisporus* cells, and enhance their resistance to external environmental stress. Moreover, this antioxidant effect indirectly inhibits microbial growth, as some oxidation by-products can serve as nutrients or promote microbial metabolism [49]. GSE has been reported to disrupt the integrity of the *E. coli* cell film, inhibit its respiratory chain and key enzyme activities, and interfere with normal metabolism [50]. Similarly, mimicking the lotus leaf structure forms a physical barrier [51]. Consistent with these findings, a CS/polyvinyl alcohol/tea polyphenol composite film exhibited antimicrobial properties, which prevented bacterial propagation [39].

#### 2.5.5. Cytotoxicity Evaluation Analysis

Through cytotoxicity assays evaluating the biocompatibility of the film, the results demonstrated that within the concentration range of 10–200 μg/mL, all film formulations exhibited high cell viability rates, indicating low cytotoxicity. Specifically, the cell viability in the BPG-40 treatment group reached 98.47%, suggesting that BPG-40 did not inhibit cell survival. These results indicate that the film extracts exhibit low in vitro cytotoxicity towards GES-1 cells under the tested conditions (10–200 μg/mL, 72 h exposure); this finding does not by itself establish general food-contact safety, which would additionally require migration testing with food simulants in accordance with applicable food-contact regulations.

**Figure 10:**
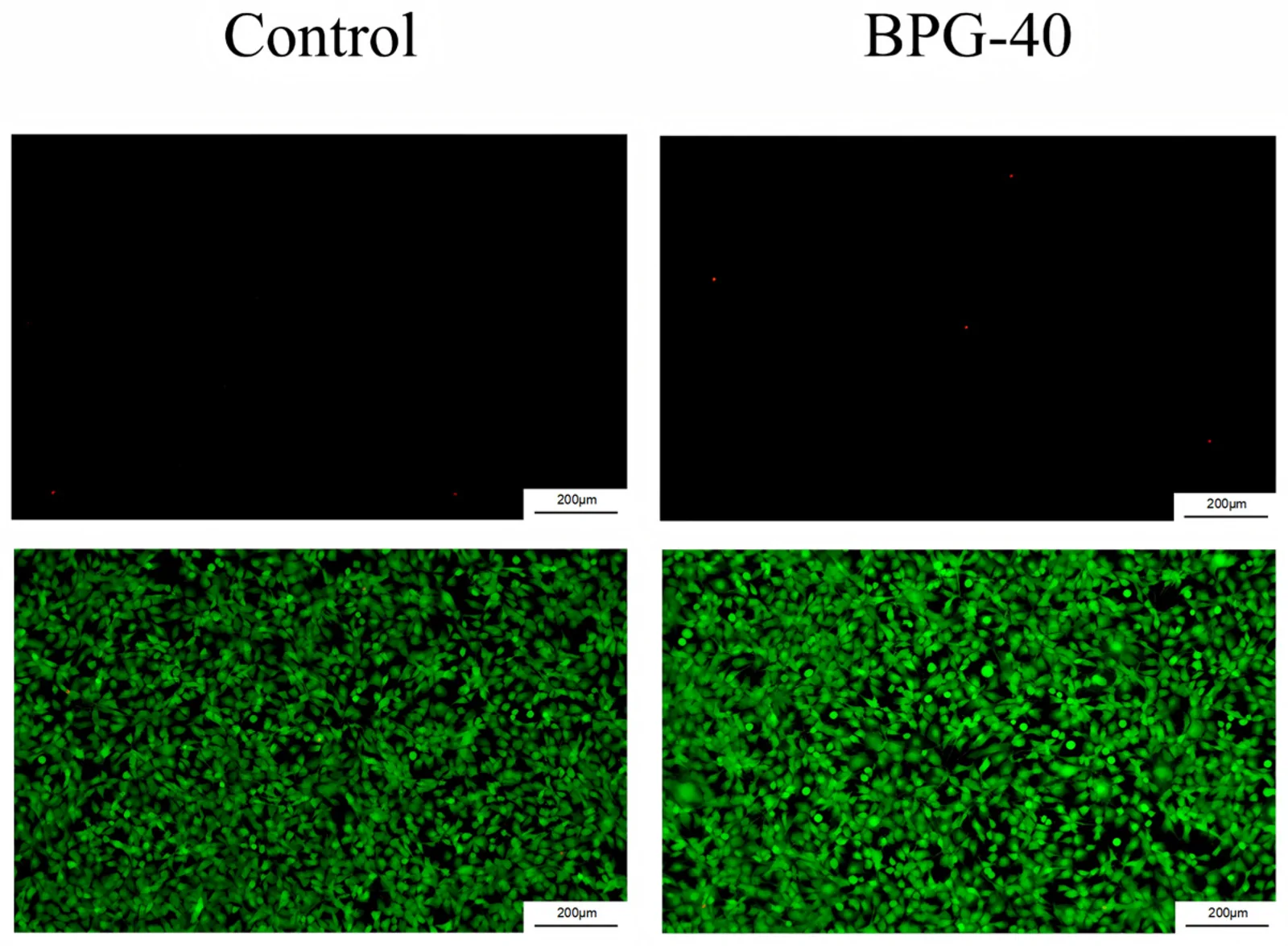
Live/Dead Cell Staining by Confocal Microscopy. Data are expressed as mean ± standard deviation (*n* = 3).

To further investigate the status of cells treated with BPG-40, live/dead cell staining was performed and visualized via confocal laser scanning microscopy, as shown in (Figure 10). The images align with the conclusions drawn from the CCK-8 cytotoxicity assay: this further validates the reliability of the CCK-8 cytotoxicity test.

### 2.6. Discussion

#### 2.6.1. Structure–Function Relationships of the Biomimetic Gel Network

As revealed by the quantitative SEM analysis (Section 2.1.1, Figure 2), casting on the lotus-leaf-templated PDMS surface consolidated the film surface: the pure PVA control showed a low-contrast surface that precluded reliable automated pore quantification (presented for morphology only in Figure 2), whereas the biomimetic BPG films showed low surface porosity (8.1–9.1%) with mean equivalent pore diameters of 7.4–8.1 μm, among which BPG-30 displayed the most homogeneous pore-size distribution. Because film thickness increased monotonically with GSE loading (0.13–0.46 mm), the tensile data were additionally compared on a thickness-normalized basis: the control film retained the highest specific tensile strength (≈176 MPa/mm), while BPG-30 (≈93 MPa/mm) clearly exceeded the other GSE-containing formulations (77–80 MPa/mm), confirming a genuine composition-dependent reinforcement at 30% loading beyond the thickness effect. These interconnected gel pores plausibly act as reservoirs for GSE and provide water-filled channels for its sustained release. The hierarchical surface structure also governs the water-absorption behaviour of the film in two coupled steps: the lotus-leaf-inspired micro/nano-protrusions delay the initial wetting of the surface and thereby slow the penetration of liquid water into the gel network, whereas the subsequent equilibrium water uptake is controlled by the density of the interior gel network—the denser, less porous network of the biomimetic films (8.1–9.1% surface porosity) restricts water uptake and underlies their lower WVP, while the open pore structure of the control film favours transient water transport. Regarding the cross-linking route, the repeated freeze–thaw process was chosen over chemical cross-linking for reasons that are directly relevant to food-contact applications: physical cross-linking through ice-crystal-induced PVA crystallization requires no external cross-linking agent, so the film contains no residual reagent (e.g., aldehyde- or borate-based) that would have to be washed out and could raise food-safety concerns. Its practical drawbacks are the longer processing time (repeated thermal cycles), the energy demand of the freezing steps, and a lower upper limit of achievable cross-linking density than chemically cross-linked analogues—an acceptable trade-off here, because the mechanical requirements of a fresh-produce packaging film are moderate.

#### 2.6.2. Rationale for Selecting BPG-30 as the Optimum Formulation

**Figure 11:**
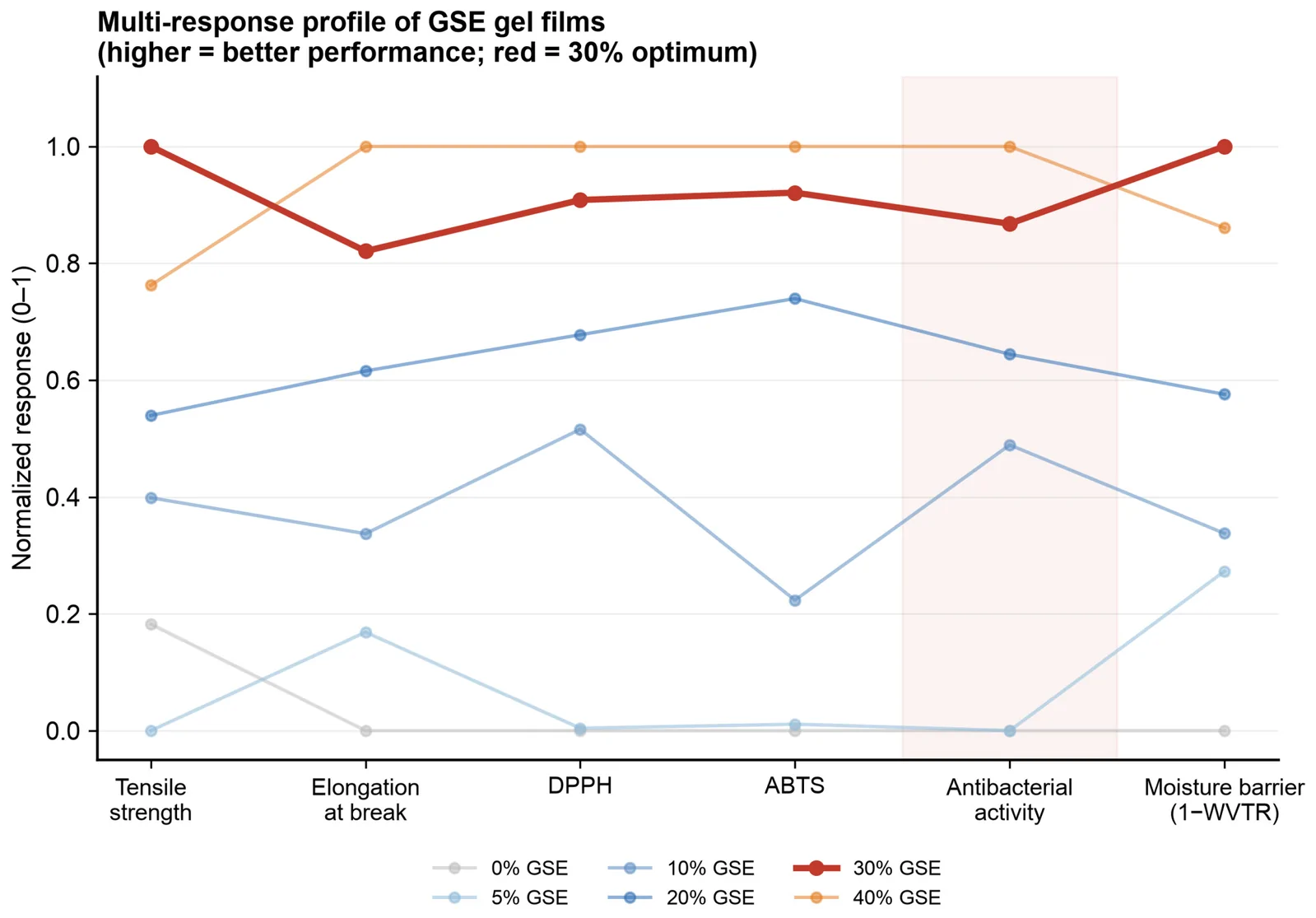
Multi-response trade-off analysis of the six film formulations across ten normalized property indicators (TS, EAB, thickness, WVP, moisture content, swelling ratio, solubility, DPPH, ABTS and antibacterial activity), highlighting the convergence of optimal responses at BPG-30.

The apparent discrepancy between the optimal film formulation (BPG-30, Abstract and Section 2.5) and the best storage treatment (BPG-40) is resolved by stating the selection criterion explicitly: the optimum formulation was selected on a multi-response balance across ten film-property indicators (TS, EAB, thickness, WVP, moisture content, swelling ratio, solubility, DPPH, ABTS and antibacterial activity; Figure 11). BPG-30 combined the highest TS (40.83 MPa), the lowest WVP (4.12 × 10^−10^ g·m^−1^·s^−1^·Pa^−1^) and the largest swelling capacity (869.62%) with 91.3% and 93.1% of the maximum DPPH and ABTS scavenging activity achieved by BPG-40. BPG-40 outperformed BPG-30 only during storage, where its higher GSE dose provided additional antioxidant and antimicrobial reserves; however, its 12.6% lower tensile strength, crack-prone surface, and near-opaque appearance (L* = 6.07, opacity 6.68 mm^−1^) make it less suitable as a practical packaging material. The two conclusions are therefore complementary rather than contradictory: BPG-30 is the optimal film formulation, whereas BPG-40 represents the upper bound of preservation performance at the cost of film quality.

#### 2.6.3. Release Behaviour and Antibacterial Mechanism

The release of GSE from the gel network is proposed to follow a two-stage mechanism: hydration-driven swelling of the network opens water-filled channels connecting the gel pores, followed by concentration-gradient-driven diffusion of the polyphenols through these channels; the hydrogen bonding between GSE polyphenols and PVA hydroxyl groups (evidenced by the FTIR shifts) retards the diffusional flux and limits burst release. The antibacterial action is dominated by the chemical activity of GSE rather than the physical biomimetic topology: the inhibition zones increased monotonically with GSE content (no inhibition for the control and BPG-0, up to 17.7 ± 0.2 mm and 20.2 ± 0.2 mm at 40% loading against *E. coli* and *S. aureus*, respectively), paralleling the dose-dependent antioxidant activity, whereas the biomimetic structure alone produced no inhibition zone, indicating at most a secondary role in preventing bacterial attachment. The mean pore diameter of ~7.4–8.1 μm measured by SEM image analysis (Figure 2) is consistent with a diffusion-controlled release profile and supports the two-stage release model described above.

#### 2.6.4. Moisture and Gas Regulation During Storage

The preservation performance can be explained by a coupled moisture–gas regulation chain: the reduced WVP of the biomimetic films attenuates water loss from the packaged mushrooms and directly limits weight loss, while the modified internal atmosphere (higher CO_2_, lower O_2_) suppresses respiration. The parallel decrease in weight loss and respiration rate during storage is consistent with this mechanism, although the headspace gas composition was not directly monitored in this study. The in-package humidity itself is not an independent parameter but the outcome of the same balance: it is set by the ratio between the moisture released by the mushroom (transpiration and respiration) and the moisture transmitted through the film. The lower WVP of the biomimetic films therefore acts on both sides of the weight-loss equation—it slows the outward moisture flux and helps maintain a higher in-package relative humidity, which in turn lowers the transpiration driving force of the mushroom. Because the headspace humidity was not monitored separately in this study, this coupled interpretation is offered as the most parsimonious explanation consistent with the parallel decreases in weight loss and respiration rate.

#### 2.6.5. Limitations

This study has several limitations that should be addressed in future work. The formulation optimization was based on single-factor screening rather than a formal design-of-experiments approach; quantitative time-resolved release kinetics of GSE were not measured; the headspace gas composition of the packages was not monitored; pore-size statistics were derived from single representative SEM fields per formulation, and nanoscale surface roughness was not quantified by AFM; the long-term stability of the GSE entrapped in the gel network (e.g., polyphenol oxidation and colour deepening during storage beyond 12 d or under light exposure) was not evaluated, although the hydrogen-bonded immobilization of GSE within the PVA network is expected to retard its degradation; and the cytotoxicity evaluation was limited to in vitro conditions, which does not by itself establish general food-contact safety.

## 3. Conclusions

In this study, we fabricated a physically cross-linked PVA gel film loaded with GSE and featuring a biomimetic lotus-leaf surface morphology via PDMS template replication and repeated freeze–thaw cycles. Compared with the pure PVA control, the optimal BPG-30 formulation increased tensile strength by 76.8% (from 23.09 to 40.83 MPa) and elongation at break by 52.9% (from 33.31% to 50.95%), reduced the water-vapour permeability by 56.5% (from 9.47 to 4.12 × 10^−10^ g·m^−1^·s^−1^·Pa^−1^), and enhanced the DPPH and ABTS radical-scavenging activities by 17.0- and 7.0-fold (to 58.61% and 60.76%), with inhibition zones of 15.36 mm (*E. coli*) and 17.82 mm (*S. aureus*) where the control showed none. During 12 days of storage at 4 °C, BPG-30 packaging reduced the weight loss of *A. bisporus* by 56.5% relative to the unwrapped group (2.92% vs. 6.72%), delayed the respiration peak from day 6 to day 9, and suppressed the accumulation of MDA and the rise in PPO activity, while BPG-40 achieved the lowest weight loss (1.95%, a 71.0% reduction) and the highest retained firmness (15 N, 40% above the unwrapped group). The film extracts showed low in vitro cytotoxicity towards GES-1 cells under the tested conditions. These quantitative results demonstrate that integrating a lotus-leaf-inspired hierarchical structure with GSE-loaded PVA gel networks is an effective strategy for active packaging of fresh produce such as *A. bisporus*.

## 4. Materials and Methods

### 4.1. Materials

GSE (95% procyanidins) was purchased from Shandong Jinsheng Biotechnology Co., Ltd. (Binzhou, China). PVA (Mw 20,000–150,000, 99% hydrolyzed) and Polydimethylsiloxane (PDMS, Sylgard 184) was obtained from Dow Corning Corporation (Midland, MI, USA). 1,1-diphenyl-2-benzhydrazine (DPPH) and 2,2-azino-4-(3-ethylbenzothiazol-6-sulfonic acid) disodium salt (ABTS) were purchased from Sigma-Aldrich (St. Louis, MO, USA). Glycerol, phosphate buffer, potassium ferricyanide, anhydrous calcium chloride, trichloroacetic acid, potassium nitrate, ferric chloride, ethanol, nutrient agar, yeast extract, and peptone were obtained from Sinopharm Chemical Reagents Co., Ltd. (Shanghai, China). *Escherichia coli* (*E. coli*) and *Staphylococcus aureus* (*S. aureus*) were provided by the Food Science and Engineering Laboratory of Jilin Agricultural University (Changchun, China). *A. bisporus* was obtained from Walmart Supermarket in Changchun, China.

### 4.2. Preparation of Films

**Figure 12:**
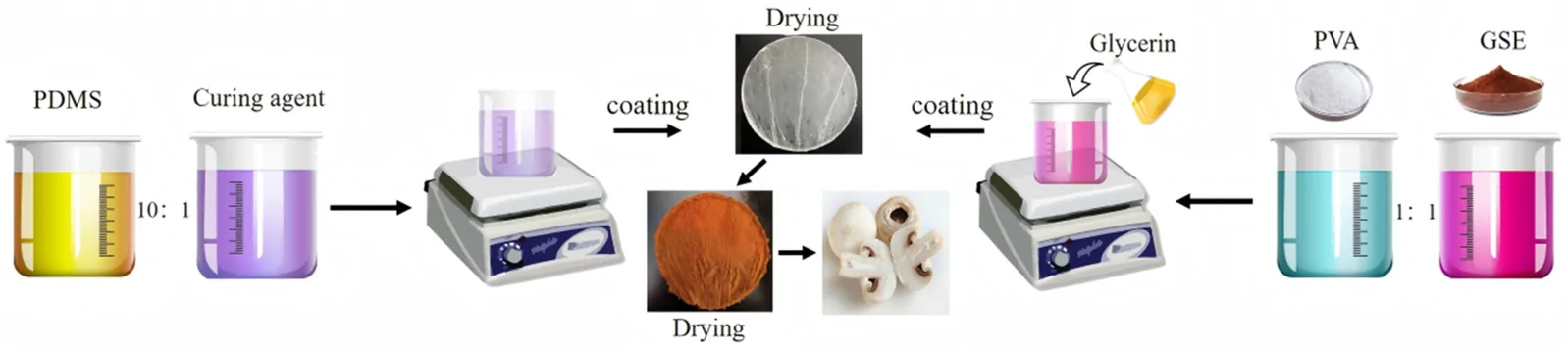
Schematic of the preparation and application of GSE–PVA bionic films.

As shown in the Figure 12, GSE was dissolved in 100 mL of distilled water to make 10%, 20%, 30%, and 40% aqueous GSE solutions. A 5% PVA solution was prepared separately, and then the GSE and PVA solutions were mixed in a 1:1 ratio. To prepare the composite film solutions, 4% glycerol was added as a plasticizer to improve the chain mobility and flexibility of the PVA–GSE matrix, and the mixture was magnetically stirred for 1 h and then left to stand for 30 min. PDMS and its curing agent were mixed at a 10:1 ratio, magnetically stirred for 2 h, and stabilized at 25 °C and 75% RH. The PDMS solution was coated on lotus leaf samples and dried at 110 °C for 1.5 h to form lotus leaf antistructured PDMS films. The composite film solution was coated onto a PDMS film, which was then frozen at −20 °C for 6 h and subsequently thawed at 25 °C under 40% relative humidity for 5 h. This cycle was repeated until the film was fully dried; the film was peeled off to obtain a bionic film featuring a leaf-like microstructure. The pure PVA film was used as the control. The biomimetic films with 0%, 10%, 20%, 30%, and 40% GSE were named BPG—0, BPG—10, BPG—20, BPG—30, and BPG—40, respectively. All of the following tests evaluate the side with a biomimetic structure.

### 4.3. Physical Properties

#### 4.3.1. Thickness Determination

The film thickness was measured at five randomly selected positions using a spiral micrometer (Suzuki GmbH, Bensheim, Germany), and the average value was calculated.

#### 4.3.2. Color Determination

The L*, a*, and b* values of the films were measured using a colorimeter (Hunter Laboratories, Reston, VA, USA). The colorimeter was calibrated with a standard whiteboard, and the color difference (ΔE) and chroma (C) were calculated as follows:

(1) $$∆E=\sqrt{{\left(∆a^{*}\right)}^{2}+(∆{b^{*})}^{2}+(∆{L^{*})}^{2}}$$

(2) $$C=\sqrt{({a^{*})}^{2}+({b^{*})}^{2}}$$

where L*, a*, and b* denote the color values of the standard whiteboard.

#### 4.3.3. Opacity Determination

According to the method of Wu et al. [52], film opacity was measured at 600 nm using a 722 UV spectrophotometer (Youke Co., Ltd., Shanghai, China), with an empty cuvette as the control. Film opacity was calculated using the following formula:

(3) $$\mathrm{opacity}=\frac{A_{600}}{d}$$

where A_600_ denotes the film absorbance at 600 nm, and d indicates the film thickness (mm).

#### 4.3.4. Moisture Content (MC) and Water Vapour Permeability (WVP)

The MC of the film was determined using the drying method. Film samples (20 × 20 mm) were first weighed to obtain the initial weight (W_0_) and then dried in an oven at 105 °C until a constant weight was reached. The dried samples were weighed (W_1_). MC was calculated using the following formula:

(4) $$\mathrm{MC}=\frac{W_{0}-W_{1}}{W_{0}}\times 100\%$$

where W_0_ denotes the initial total mass, and W_1_ indicates the final mass (g).

The WVP of the film was measured and modified according to the method of Mo et al. [53]. A 20 × 20 mm film was placed over a weighing cup (40 mm diameter) containing anhydrous CaCl_2_. The cup was then kept in a desiccator with a saturated KNO_3_ solution, and its weight was recorded every 12 h for 5 days. WVP was calculated using the following formula:

(5) $$\mathrm{WVP}=\frac{∆\mathrm{wd}}{S\Delta t∆P}$$

where Δw indicates the weight change in the cup (g); d denotes the film thickness (mm); S represents the area of the sealed film (m_2_); Δt indicates the time interval (h); and ΔP signifies the vapor pressure difference across the film (kPa).

#### 4.3.5. Oxygen Permeability (OP) and Carbon Dioxide Permeability (CDP)

According to our previous report, the OP and CDP of the films were determined using the deoxidizer absorption and potassium hydroxide methods, respectively [54]. For OP measurement, a deoxidizer was placed in a weighing bottle and sealed with a circular film (radius 2.0 cm) using an elastic band. The sealed bottle was placed in a drying chamber maintained at 25 °C and 75% RH for 48 h. The OP value was calculated using the corresponding equation. For CDP measurement, 3 g of KOH was placed in a weighing bottle and sealed with a circular film under the same environmental conditions. The CDP value was determined from the recorded data.

OP = (Δm × d)/(A × t × P)(6)

CDP = (Δm × d)/(A × t × P)(7)

where Δm denotes the change in test bottle weight (kg), d indicates the film thickness (m), A signifies the effective area of the film (m^2^), t represents the test duration (s), and P denotes the partial pressure difference in the test gas across the film: for OP measurement, P(O_2_) ≈ 0.21 atm (2.13 × 10^4^ Pa), since the internal O_2_ is consumed by the deoxidizer while the exterior is air; for CDP measurement, P(CO_2_) ≈ 4.05 × 10^−4^ atm (41 Pa) in normal atmosphere, since CO_2_ is absorbed by the KOH inside the bottle. The humidity (75% RH) was controlled to keep the films at a comparable hydrated state, but water vapour was not the driving gas in these measurements. All OP and CDP measurements were performed in triplicate.

#### 4.3.6. Mechanical Properties Determination

The mechanical strength of the film was determined according to the method of Wu et al. Tensile strength (TS) and elongation at break (EAB) were measured using an electronic universal testing machine [55]. Film samples were cut into strips of 60 × 20 mm. Tests were conducted at a crosshead speed of 50 mm/min. Each sample was tested in triplicate. TS and EAB were calculated using the following formula:

(8) $$\mathrm{TS}(\mathrm{MPa})\;=\;\frac{F_{\max}}{d\;\times \;w}$$

(9) $$\mathrm{EAB}(\%)=\frac{L_{I}-L_{0}}{L_{0}}$$

where F_max_ denotes the maximum force required to break the film (N); w indicates the film width (mm); L_1_ denotes the film length at fracture (mm); L_0_ indicates the original length of the film (mm).

### 4.4. Structural Characterization Determination

#### 4.4.1. Scanning Electron Microscopy (SEM) Analysis of the Films

The surface microstructure of the films was examined via SEM (DSF-60S-MS, Su Liang Instruments Co., Ltd., Suzhou, China) at magnifications of 500–3000× and a resolution range of 2–100 μm [56].

#### 4.4.2. Fourier Transform Infrared Spectroscopy (FT-IR) Analysis of the Films

The FTIR spectra of the films were recorded using an FTIR spectrometer (Nicolet iS50, Thermo Fisher Scientific, Waltham, MA, USA) equipped with ATR accessories, over the 400–4000 cm^−1^ range, with 64 scans at 4 cm^−1^ resolution [30,57,58].

#### 4.4.3. X-Ray Diffraction (XRD) Analysis of the Films

The crystallinity of the films was measured using an Empyrean X-ray diffractometer (Thermo Fisher Scientific, Shanghai, China) at 40 kV and 40 mA. Films were scanned over a 2*θ* range of 5–40° at 2°/min. The XRD patterns were recorded.

### 4.5. Antioxidant Properties

#### 4.5.1. DPPH

The DPPH free radical scavenging activity of the films was determined according to the method of Kaya et al. [59], with slight modifications. The films were dissolved in 4 mL of ethanol, and 1.5 mL of this solution was mixed with 2 mL of 0.1 mM DPPH in ethanol. The mixture was incubated in the dark at 25 °C for 30 min. The absorbance of the reaction mixture (A_0_) and the film extract (A_1_) was measured at 517 nm. The DPPH radical scavenging rate was calculated as follows:

(10) $$\mathrm{DPPH}\;\mathrm{free}\;\mathrm{radical}\;\mathrm{scavenging}\;\mathrm{rate}\;(\%)\;=\;\frac{A_{0}-A_{1}}{A_{0}}\times 100$$

where A_0_ denotes the absorbance of the blank control, and A_1_ indicates the absorbance of the sample.

#### 4.5.2. ABTS

The ABTS free radical scavenging activity of the films was determined according to the method of Constantino et al. [60], with slight modifications. A 7 mmol/L ABTS ethanol solution and a 2.45 mmol/L aqueous potassium persulfate solution were mixed at a 1:1 ratio to prepare the ABTS stock solution. The mixture was left in the dark for 24 h to form ABTS+. The solution was diluted with ethanol to obtain the appropriate absorbance at 734 nm. Subsequently, 50 μL of film solution was added to 200 μL of ABTS solution, and absorbance was measured at 0 and 30 min, denoted as A_0_ and A_1_, respectively. The ABTS radical scavenging rate was calculated using the following formula. Each experiment was performed in triplicate:

(11) $$\mathrm{ABTS}\;\mathrm{radical}\;\mathrm{scavenging}\;\mathrm{rate}\left(\%\right)=\frac{A_{0}-A_{1}}{A_{0}}\times 100$$

#### 4.5.3. Ferric Reducing Antioxidant Power (FRAP)

First, 0.5 mL of film extract, 0.2 mL of 0.2 mol/L phosphate-buffered solution (PBS, pH 6.6), and 0.5 mL of 1% potassium ferricyanide solution were thoroughly mixed. The mixture was incubated in a water bath at 50 °C for 20 min, followed by the addition of 1 mL of 10% trichloroacetic acid. The solution was centrifuged at 1509 *g* for 10 min. Afterward, 1.5 mL of the supernatant was mixed with 1 mL of 1% ferric chloride and 3 mL of distilled water and allowed to stand for 10 min. The absorbance was measured at 700 nm.

### 4.6. Antibacterial Properties

*E. coli* and *S. aureus* were used to evaluate the antimicrobial activity of the biomimetic films according to the method of Li et al. [61], with slight modifications. A bacterial suspension (10^5^ CFU/mL) was evenly spread on an agar plate, and a circular film (10 mm in diameter) was placed on the agar surface. The plate was incubated at 37 °C for 12 h, and the size of the inhibition zone was measured. All experiments were performed in triplicate.

### 4.7. Application Determination

The prepared biomimetic films were used to preserve *A. bisporus* and evaluate their effects on *A. bisporus* quality. Fresh *A. bisporus* of similar size and maturity, free from pests, diseases, and mechanical damage, were selected from a supermarket. The *A. bisporus* were individually packed in BPG-0, BPG-10, BPG-20, BPG-30, and BPG-40 films with the biomimetic structure facing upward or the smooth surface contacting the *A. bisporus* surface; an unwrapped group exposed to air was also established. Each treatment group comprised fifteen packages, each containing one *A. bisporus* fruiting body, with a package size of 80 × 80 mm (effective film area A = 64 cm^2^); the ratio of the film area to the mushroom volume is approximately 100:31. All samples were stored at 4 ± 1 °C for 12 d. On each sampling day (0, 3, 6, 9 and 12 d), three packages were randomly collected for analysis.

Throughout this study, the following terminology is used consistently: (i) Control refers to the pure PVA film cast on a flat plate (no lotus-leaf template, no GSE), used only in film-characterization experiments; (ii) BPG-0 refers to the biomimetic PVA film cast on the lotus-leaf-templated PDMS surface without GSE; (iii) BPG-10/20/30/40 refer to biomimetic PVA films containing the corresponding GSE loadings (10–40%); and (iv) Unwrapped refers to the storage group in which *A. bisporus* was stored without any packaging film. In the storage experiment, all packaged groups are compared against this unwrapped group.

#### 4.7.1. Physical Characteristics of *A. bisporus* Determination

During the experiment, photographs were captured to record changes in *A. bisporus*, such as color, cap opening, odor, and texture. The determination of sensory quality was performed using the method described by Wang [47]. This sensory evaluation was conducted by 10 laboratory staff members from the College of Food Science at Jilin Agricultural University (Changchun, China) who had received standardized training. The assessment was completed under natural light conditions at 25 °C and 75% humidity. All evaluators provided informed consent, and the study complied with ethical standards. The attributes evaluated were colour, taste, texture and overall acceptability, and the total sensory score was the sum of all attribute scores (maximum 50 points); a total score below 30 was defined as the limit of marketability. Samples from all treatment groups were presented simultaneously in random order and labelled with three-digit random codes; the assessors were blinded to the treatment allocation and evaluated the samples individually, with water provided for palate cleansing.

#### 4.7.2. Weight Loss

The weight loss of *A. bisporus* was determined, and measurements were performed in triplicate for each sample. The weight loss was calculated using the following formula [54]:

(12) $$\mathrm{Weight}\;\mathrm{loss}\;\left(\%\right)\;=\;(\frac{W_{0}-W_{n}}{W_{0}})\;\times \;100$$

where W_0_ denotes the initial weight of *A. bisporus*, and W_n_ indicates the weight on the nth day of storage (g).

#### 4.7.3. Firmness

The firmness of *A. bisporus* was measured using a texture analyzer (TA-XT, Stable Micro Systems, Godalming, England). The results were recorded in Newtons (N).

#### 4.7.4. Polyphenol Oxidase (PPO) Activity

PPO activity was determined according to the method of Xu et al. [62], with slight modifications. First, 1 g of *A. bisporus* tissue was ground and mixed with a phosphate buffer containing 5% *w*/*v* polyvinyl pyrrolidone in an ice bath. The mixture was then centrifuged at 4 °C and 16,770 *g* for 20 min. The resulting supernatant was collected and filtered to obtain the crude enzyme solution. For the assay, 0.1 mL of the enzyme extract was added to 4 mL of 0.1 mM PBS (pH 7.0) and 0.5 mL of 0.1 mol L^−1^ catechol. Absorbance at 410 nm was recorded at 30 s intervals. PPO activity was expressed in U/g and calculated using the following formula. All measurements were performed in triplicate.

(13) $$\mathrm{PPO}\;\mathrm{activity}\left(U/g\right)=\frac{\Delta A_{410}\times V}{0.01\times m\times V_{1}}\times 100$$

where ΔA_410_ denotes the change in absorbance per min; V indicates the total volume of the sample extract; m denotes the mass of *A. bisporus*; V_1_ denotes the volume of the crude enzyme solution used in the assay; 0.01 signifies a conversion factor used to standardize the enzyme activity units (U/g).

#### 4.7.5. Malondialdehyde (MDA) Content

The MDA content was measured using the thiobarbituric acid (TBA) colorimetric method. First, 2 g of *A. bisporus* was mixed with 8 mL of 10% aqueous trichloroacetic acid (TCA) solution and centrifuged at 4193 *g*/min for 12 min. The supernatant was then combined with an equal volume of TBA solution (1:1) and heated in a water bath for 15 min. After cooling to 25 °C, the absorbance was recorded at 532 nm, 600 nm, and 450 nm. The MDA content was calculated using the following formula.

(14) $$\mathrm{MDA}\;(\mu \mathrm{mol}{/}^{{\mathrm{kg}}^{-1}}(\mathrm{FW}))=\frac{{[6.45\;(A}_{532}\;-\;A_{600})-0.56A_{450}]\;\times \;8\;\times \;2}{W_{F}}$$

where 6.45 and 0.56 represent correction coefficients used to eliminate sugar interference; A_532_, A_600_, and A_450_ denote the absorbance of the reaction solution at 532 nm, 600 nm, and 450 nm, respectively; 8 signifies the volume of 10% TCA (mL); 2 denotes the conversion factor from grams to kilograms; W_F_ represents the sample weight.

#### 4.7.6. Total Phenolic Content (TPC)

TPC was determined using the Folin–Ciocalteu method with slight modifications. First, 5 g of *A. bisporus* was homogenized in 20 mL of 80% ethanol and centrifuged at 4193 *g* for 10 min. The collected supernatant (1.0 mL) was thoroughly mixed with 0.1 mL of 10% Folin reagent and incubated in the dark for 5 min. Subsequently, 5.0 mL of Na_2_CO_3_ solution was added, and the mixture was kept in the dark for 2 h. Absorbance was measured at 750 nm, and TPC was calculated using a gallic acid standard curve.

(15) $$\mathrm{TPC}\left(\mathrm{mg}/g\right)=\frac{c\times V}{m\times V_{1}}\times 100$$

where c denotes the mass of gallic acid obtained from the standard curve; V indicates the total volume of the extraction solution; m represents the mass of *A. bisporus*; V_1_ signifies the volume of the supernatant used for the measurement.

#### 4.7.7. Microbiological Analysis

The antibacterial activity of the films was evaluated by the agar diffusion method. Briefly, the bacterial suspension was evenly spread onto the surface of the agar plate. The film samples were cut into uniform pieces of a specified size/diameter and sterilized before use. Each sample film was then placed directly on the inoculated agar surface and gently pressed to ensure full contact with the medium. The plates were incubated at 37 °C for 24 h. After incubation, the inhibition zones around the samples were photographed and the diameter of the clear zone was measured with a ruler/caliper. All tests were performed in triplicate.

#### 4.7.8. Cytotoxicity Evaluation

Film extracts were prepared by dissolving sterile film discs in RPMI-1640 medium supplemented with 10% FBS at 37 °C for 24 h, and serially diluted to nominal concentrations of 10–200 μg/mL before administration to the cells.

GES-1 cell suspension (cell density of 4 × 10^3^ cells/mL) of 100 μL was seeded into 96-well culture plates and incubated in a 37 °C, 5% CO_2_ incubator for 24 h. After incubation, the original RPMI-1640 medium supplemented with 10% FBS was removed, and 100 μL of film solutions at different concentrations were added to each well, followed by continued incubation for 72 h. Upon completion of incubation, 10 μL of CCK-8 and 90 μL of fresh RPMI-1640 medium supplemented with 10% FBS were added to each well. After thorough mixing, the plates were incubated in the incubator for 2 h, and then the absorbance at 450 nm wavelength was measured.

(16) $$\mathrm{Relative}\;\mathrm{viability}\left(\%\right)=\frac{{\mathrm{OD}}_{\mathrm{BPG}\text{-}40}-{\mathrm{OD}}_{\mathrm{blank}}}{{\mathrm{OD}}_{\mathrm{control}}-{\mathrm{OD}}_{\mathrm{blank}}}\times 100$$

where *OD_BPG_*_-40_, *OD_control_*, and *OD_blank_* correspond to the absorbance readings at 450 nm for the test sample, positive control, and blank control, respectively.

GES-1 cells in logarithmic growth phase were quantified and seeded at a density of 1 × 104 cells per confocal dish, followed by overnight incubation at 37 °C with 5% CO_2_. The blank control group received 1000 μL of complete medium, while the BPG-40 group was treated with 1000 μL of working solution. Both groups were subsequently cultured for 72 h, with three replicates per group. Calcein-AM and PI were diluted 10-fold using solution C. A staining solution was prepared by combining 985.5 μL of PBS with 10 μL of diluted Calcein-AM and 4.5 μL of PI solution. After washing once with PBS, cells were incubated with 1 mL of staining solution under dark conditions for 15 min, followed by three PBS washes prior to observation and imaging.

#### 4.7.9. Determination of Total Colony Counts

First, 5 g of *A. bisporus* was added to 45 mL of sterile 0.1% peptone water and thoroughly mixed. Serial dilutions (10^−1^ to 10^−9^) were prepared, and 1 mL of each dilution was plated. Colony counts were expressed as CFU/g. Each group was prepared in triplicate.

#### 4.7.10. Statistical Analysis

All film-characterization experiments were performed on three independently prepared film batches (*n* = 3 biological replicates), with technical measurements taken in triplicate within each batch. For the storage experiment, each treatment comprised fifteen packages (one *A. bisporus* fruiting body per package, 80 × 80 mm); on each sampling day (0, 3, 6, 9 and 12 d), three packages were randomly collected and analysed, and the reported values are the mean ± SD of three biological replicate packages with technical triplicates per package. The statistical unit is the individual film batch for film data and the individual package for storage data. Multiple comparisons were performed using SPSS 26.0, and plots were generated using Origin 2019.

## Figures and Tables

**Table 1:** Color values of PVA-GSE bionic films.

Films	L*	a*	b*	ΔE*	C*	Opacity (Amm^−1^)
Control	85.57 ± 0.28 ^a^	0.62 ± 0.34 ^e^	44.45 ± 0.78 ^a^	51.17 ± 0.53 ^d^	44.45 ± 0.78 ^a^	0.76 ± 0.13 ^a^
BPG-0	83.29 ± 0.61 ^a^	1.52 ± 0.25 ^e^	44.43 ± 0.58 ^a^	51.47 ± 0.32 ^d^	44.45 ± 0.58 ^a^	1.24 ± 0.26 ^a^
BPG-10	16.18 ± 0.88 ^b^	6.08 ± 0.83 ^d^	4.83 ± 0.74 ^b^	49.22 ± 1.02 ^e^	7.77 ± 1.03 ^e^	2.00 ± 0.32 ^b^
BPG-20	12.86 ± 0.42 ^c^	9.70 ± 1.08 ^c^	3.81 ± 0.42 ^c^	54.21 ± 0.72 ^c^	10.43 ± 1.04 ^d^	3.29 ± 0.24 ^c^
BPG-30	9.45 ± 0.27 ^d^	11.79 ± 1.10 ^b^	3.64 ± 0.49 ^c^	58.10 ± 0.80 ^b^	12.35 ± 1.07 ^c^	5.43 ± 0.18 ^d^
BPG-40	6.07 ± 0.66 ^e^	15.48 ± 1.07 ^a^	0.34 ± 0.10 ^d^	63.66 ± 0.83 ^a^	15.49 ± 1.07 ^b^	6.68 ± 0.57 ^e^

Note: Different lowercase letters after the results represent significant differences under the same column (*p* < 0.05). Data are expressed as mean ± standard deviation (*n* = 3); The asterisk (*) denotes the CIE standard notation for color parameters (L*, a*, b*, E*, C*). Control was used as the reference group with ΔE = 0 and was excluded from intergroup statistical comparisons.

**Table 2:** Moisture content, WVP, Water solubility, Swelling ratio and Thickness of PVA-GSE bionic films.

Films	Moisture Content (%)	WVP (10^−10^ g m^−1^ s^−1^ Pa^−1^)	Water Solubility (%)	Swelling Ratio (%)	Thickness (mm)
Control	18.44 ± 0.11 ^a^	9.47 ± 1.39 ^a^	16.14 ± 0.70 ^a^	517.88 ± 0.48 ^a^	0.13 ± 0.01 ^a^
BPG-0	13.31 ± 0.42 ^b^	8.01 ± 0.44 ^b^	18.11 ± 0.18 ^b^	553.33 ± 0.50 ^b^	0.24 ± 0.01 ^b^
BPG-10	10.72 ± 0.10 ^c^	7.67 ± 0.23 ^b^	22.83 ± 0.56 ^c^	649.32 ± 1.01 ^c^	0.36 ± 0.03 ^c^
BPG-20	9.65 ± 0.26 ^d^	6.39 ± 0.40 ^c^	22.83 ± 0.50 ^c^	736.24 ± 0.68 ^d^	0.39 ± 0.03 ^c^
BPG-30	8.63 ± 0.21 ^e^	4.12 ± 0.19 ^d^	27.63 ± 0.24 ^d^	869.62 ± 0.43 ^e^	0.44 ± 0.01 ^d^
BPG-40	7.70 ± 0.31 ^f^	4.87 ± 0.15 ^d^	30.24 ± 0.62 ^e^	901.67 ± 0.65 ^e^	0.46 ± 0.04 ^d^

Note: Different lowercase letters after the results represent significant differences under the same column (*p* < 0.05). Data are expressed as mean ± standard deviation (*n* = 3).

**Table 3:** TS and EAB of PVA-GSE bionic films.

Films	TS (MPa)	EAB (%)
Control	23.09 ± 0.42 ^b^	33.31 ± 0.65 ^a^
BPG-0	19.13 ± 0.73 ^a^	36.93 ± 0.63 ^b^
BPG-10	27.78 ± 0.40 ^c^	40.56 ± 0.77 ^c^
BPG-20	30.84 ± 0.88 ^d^	46.54 ± 1.20 ^d^
BPG-30	40.83 ± 0.62 ^f^	50.95 ± 0.50 ^e^
BPG-40	35.67 ± 0.40 ^e^	58.80 ± 0.85 ^f^

Note: Different lowercase letters after the results represent significant differences under the same column (*p* < 0.05). Data are expressed as mean ± standard deviation (*n* = 3).

**Table 4:** Antioxidant properties of PVA-GSE bionic films.

Films	DPPH Free Radical Scavenging Activity (%)	ABTS Free Radical Scavenging Activity (%)	FRAP (mg)
Control	3.44 ± 0.08 ^e^	8.69 ± 0.37 ^e^	0.47 ± 0.04 ^e^
BPG-0	3.70 ± 0.37 ^e^	9.31 ± 0.11 ^e^	0.50 ± 0.04 ^e^
BPG-10	34.77 ± 0.51 ^d^	21.32 ± 0.39 ^d^	0.91 ± 0.09 ^d^
BPG-20	44.60 ± 0.38 ^c^	50.51 ± 0.69 ^c^	1.42 ± 0.07 ^c^
BPG-30	58.61 ± 0.25 ^b^	60.76 ± 0.66 ^b^	1.59 ± 0.03 ^b^
BPG-40	64.18 ± 0.18 ^a^	65.24 ± 0.35 ^a^	1.83 ± 0.04 ^a^

Note: The different lowercase letters after the results represent significant differences within the same column (*p* < 0.05). Data are expressed as mean ± standard deviation (*n* = 3).

**Table 5:** Antibacterial activity of PVA-GSE bionic films.

Films	Inhibition Zone of *E. coli* (mm)	Inhibition Zone of *S. aureus* (mm)
Control	—	—
BPG-0	—	—
BPG-10	8.66 ± 0.34 ^a^	9.65 ± 0.24 ^a^
BPG-20	11.40 ± 0.43 ^b^	14.88 ± 0.08 ^b^
BPG-30	15.36 ± 0.30 ^c^	17.82 ± 0.14 ^c^
BPG-40	17.70 ± 0.16 ^d^	20.16 ± 0.23 ^d^

Note: Different lowercase letters after the results represent significant differences under the same column (*p* < 0.05). Data are expressed as mean ± standard deviation (*n* = 3).

## Data Availability

The original contributions presented in this study are included in the article/Supplementary Materials. Further inquiries can be directed to the corresponding authors.

## Supplementary Materials

The following supporting information can be downloaded at: https://www.mdpi.com/article/10.3390/gels12100881/s1, Figure S1: Live/dead staining of GES-1 cells cultured with BPG-40 extract for 24 h; Figure S2: (A) Lotus leaf structure; (B) PDMS reverse-structured film; Figure S3: Schematic diagram of freshening packaging for double-spore *A. bisporus*; Figure S4: The OP and CDP values of the samples, Control, BPG-0, BPG-10, BPG-20, BPG-30, and BPG-40 film.
