# Supplementary material for: Preparation of a Lotus-Leaf-Inspired PVA–Grape Seed Extract Biomimetic Gel Film and Its Application in Active Packaging for the Postharvest Preservation of Agaricus bisporus
Source: Gels. 2026 Sep 30;12(10):881. doi: 10.3390/gels12100881 (PMC13704083; doi:10.3390/gels12100881)
Supplement: Supplementary file 1 [file gels-12-00881-s001.zip › gels-4546119-supplementary.pdf]

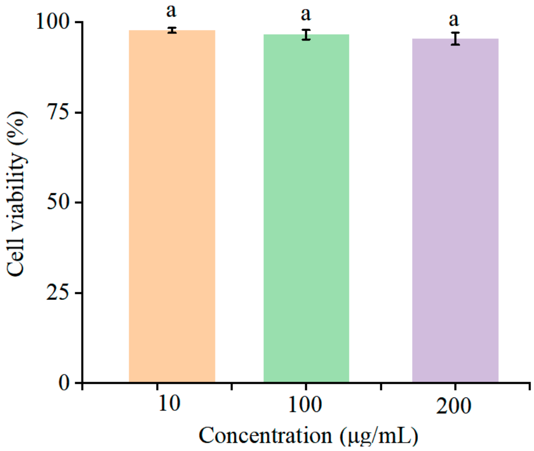

Figure S1 Live/dead staining of GES-1 cells cultured with BPG-40 extract for 24h .

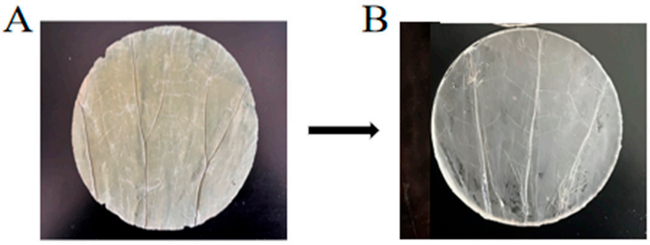

Figure S2 (A) Lotus leaf structure; (B) PDMS reverse-structured film .

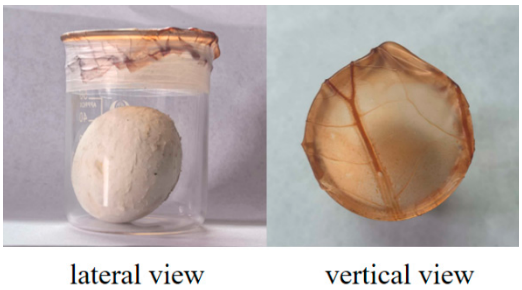

Figure S3 Schematic diagram of freshening packaging for double-spore *A. bisporus*

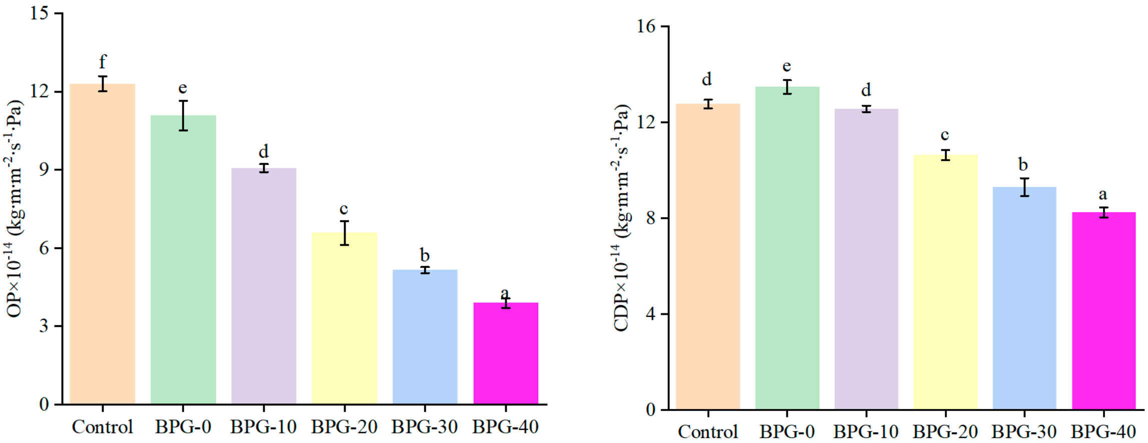

Figure S4 The OP and CDP values of the samples, Control, BPG-0, BPG-10, BPG-20, BPG-30, and BPG-40 film .
